# Temporal transcriptome and WGCNA analysis unveils divergent drought response strategies in wild and cultivated *Solanum* varieties

**DOI:** 10.3389/fpls.2025.1572619

**Published:** 2025-07-30

**Authors:** Athira C. Rajeev, Rajesh Raju, Archana Pan

**Affiliations:** ^1^ Department of Bioinformatics, School of Life Sciences, Pondicherry University, Puducherry, India; ^2^ Centre for Integrative Omics Data Science (CIODS), Yenepoya (Deemed to be University), Mangalore, India

**Keywords:** *Solanum*, wild, cultivated, drought, transcriptome, WGCNA

## Abstract

Drought, exacerbated by climate change, threatens global food security, particularly impacting crop products, including tomatoes, which are economically essential but drought sensitive. This study explores drought responses in the wild-type *Solanum pennellii* (WT), known for drought tolerance, and cultivated *Solanum lycopersicum* (CT), through RNA-Seq analysis at three drought intervals (2 Weeks +5D, +8D, and +11D). Across these points, WT and CT showed 716 and 1459 differentially expressed genes (DEGs), respectively. Pathway enrichment revealed distinct metabolic adaptations: wild varieties prioritized arginine and proline metabolism early, shifting to cutin, suberin, and wax biosynthesis by day 11, while cultivated varieties emphasized steroid biosynthesis, secondary metabolite production, and photosynthesis-related pathways. Transcription factor analysis highlighted HB-HD-ZIP enrichment in wild varieties, contrasting with broader, less coordinated TF activation in cultivated varieties. WGCNA identified the blue module as significantly associated with prolonged drought in both species. Network analysis showed ribosomal pathways enriched in CT, while in WT, it was observed broader pathway enrichment, including secondary metabolites, carbon metabolism, and oxidative stress pathways. In WT unique hub genes were, sucrose synthase and malate synthase, suggesting specialized drought adaptation mechanisms. These findings highlight multifaceted drought resilience strategy of WT compared to growth-focused response of CT, offering a foundation for breeding drought-resistant tomato varieties critical for food security under climate pressures.

## 1 Introduction

*Solanum lycopersicum L.* (tomato) is an economically important and nutritionally valuable crop worldwide, with 189 million tons cultivated on 5.16 million hectares, according to the (Food and Agriculture Organization of the United Nations The FAO Statistical Database-Agriculture. 2020 (http://faostat3.fao.org/faostatgateway/go/to/download/Q/QC/E)). This translates to roughly 170 million tons reaching consumers annually, making it one of the world’s most important food crops. In exports, tomato plants are exported to a variety of countries, helping to maintain trade balances and boosting the economies of the producing countries. The United States, China, Turkey, and Egypt are among the major tomato-producing countries. Tomatoes are rich in essential nutrients, including alpha/beta-carotene, lycopene, tomatine, and esculeoside A, contributing to human health by reducing the risk of chronic diseases like cardiovascular disease (Huang et al., 2021), cancers (Middha et al., 2019), and diabetes (Zhu et al., 2020). The increasing challenges due to climate change, particularly the rising frequency, and intensity of drought events, threaten the global production of tomatoes.

From a meteorological perspective, drought is a period of deficient rainfall compared to historical averages. This lack of precipitation disrupts the water balance in agricultural and natural ecosystems, hindering plant growth and productivity (Krishna et al., 2022). It affects the crops by generating reactive oxygen species (ROS), which disturbs cellular homeostasis (Krishna et al., 2021). Antioxidant defense and osmotic regulation are the major strategies used by plants to combat drought. Other schemes include alterations in the expression of ROS scavenging enzymes, (LEA)/dehydrin-type genes, and the synthesis of molecular chaperones, proteinases, and other detoxification proteins (Rai et al., 2013). During drought stress, tomatoes experience a surge in a plant hormone called abscisic acid (ABA) (Vishwakarma et al., 2017). This rise in ABA coincides with significant changes in gene expression patterns within their leaves. Members of the abscisic-acid-responsive element binding protein (AREB)/abscisic acid element binding factor (ABF) subfamily of basic leucine zipper (bZIP) transcription factors have been involved in the response to ABA and abiotic stress. Proline (Ghosh et al., 2022) is also known to confer better drought-withstanding capability to plants. Similarly, jasmonic acid (Raza et al., 2021), carotenoid biosynthesis (Khoo et al., 2011), and catalases, glutathione reductase, and superoxide dismutases contribute to a holistic regulation of drought stress response (Sarker and Oba, 2018).

Widely reported transcription factors (TFs) expressed during drought stress are Myb (Baldoni et al., 2015), bHLH (Liang et al., 2022), AP2/ERF (Xu et al., 2011), and WRKY (Genome wide expression analysis of WRKY genes in tomato (Solanum lycopersicum) under drought stress, 2018). Numerous reviews highlighting the role of different TFs in various biotic and abiotic stresses in plants like *Arabidopsis, Oryza sativa, Nicotiana tabacum and Zea mays* (Liu et al., 2024) underline the importance of these regulators in controlling drought. Integrating drought-stress-tolerant genes from other species into tomatoes has been the focus of intense research efforts from scientists worldwide. Some of the transgenes used for improving drought tolerance include osmotin (to improve stress response) (Goel et al., 2010), CBF1 (a stress-inducing transcription factor) (Hsieh et al., 2002)*, LeNCED1* (to increase abscisic acid (ABA) accumulation) (Wu et al., 2007). Studies have shown success in developing transgenic tomatoes with improved drought tolerance even better than wild types. For example, introducing the *AtCBF1* gene in cultivated varieties resulted in significantly higher yields than wild-type tomatoes under drought stress (Ratcliffe and Riechmann, 2002).

Wild tomato species, such as *Solanum pennellii* and *Solanum pimpinellifolium*, have evolved in diverse ecological niches, exhibiting natural adaptations to withstand water scarcity. *Solanum pennellii* (Bolger et al., 2014) and *Solanum pimpinellifolium* (Razali et al., 2018), exhibit remarkable resilience to various environmental stresses, including drought. Unlike cultivated varieties, *S. pennellii* thrives with minimal water. This translates to superior water-use efficiency and resistance to wilting, allowing it to flourish even in dry conditions. Towards this end, harnessing the genetic diversity in wild tomato varieties known for their drought resistance and adaptations to arid environments, emerges as a promising approach for improving drought tolerance in cultivated varieties.Studies like (Tirnaz et al., 2022) discuss the effectiveness and resources of using wild varieties in enhancing various traits. Traditional breeding approaches have made considerable strides in developing drought-resistant cultivars, yet the genetic potential within wild tomato relatives remains a largely untapped resource. Although efforts are being undertaken to develop drought-tolerant varieties, the rate of growth is very slow. Genetic variations in tomatoes and the multigenic nature of drought tolerance further complicate the rate.

RNA sequencing (RNA-Seq) has revolutionized plant science research, offering a powerful approach to understanding gene expression and improving crop traits. It aids in breeding by identifying candidate genes, understanding gene regulation, characterizing genetic variations, abiotic stress responses, metabolic pathway analysis, and many more. (Liu et al., 2021) Reported the identification of candidate genes for drought tolerance in maize seedlings, similarly (Pereyra-Bistraín et al., 2021), reported the identification of bacterial canker resistance genes in tomatoes using RNA-seq. Genes for resistance to Tomato Leaf Curl New Delhi Virus were identified in melon by (Sáez et al., 2021). Transcriptome profiling reveals the expression and regulation of genes associated with Fusarium wilt resistance in chickpeas (Garg et al., 2023).

In this context, here, we analyzed the time series drought-response transcriptome data of resistant and susceptible *Solanum* varieties for the study. The differentially expressed genes were identified and functionally annotated. Pathway enrichment analysis, and transcription factor analysis were performed on identified DEGs of both species. Additionally, WGCNA was conducted to identify significant modules associated with drought in 2 weeks+11D, construct a PPI network, and uncover key pathways involved in drought response.

## 2 Materials and methods

### 2.1 Data collection

**Figure 1:**
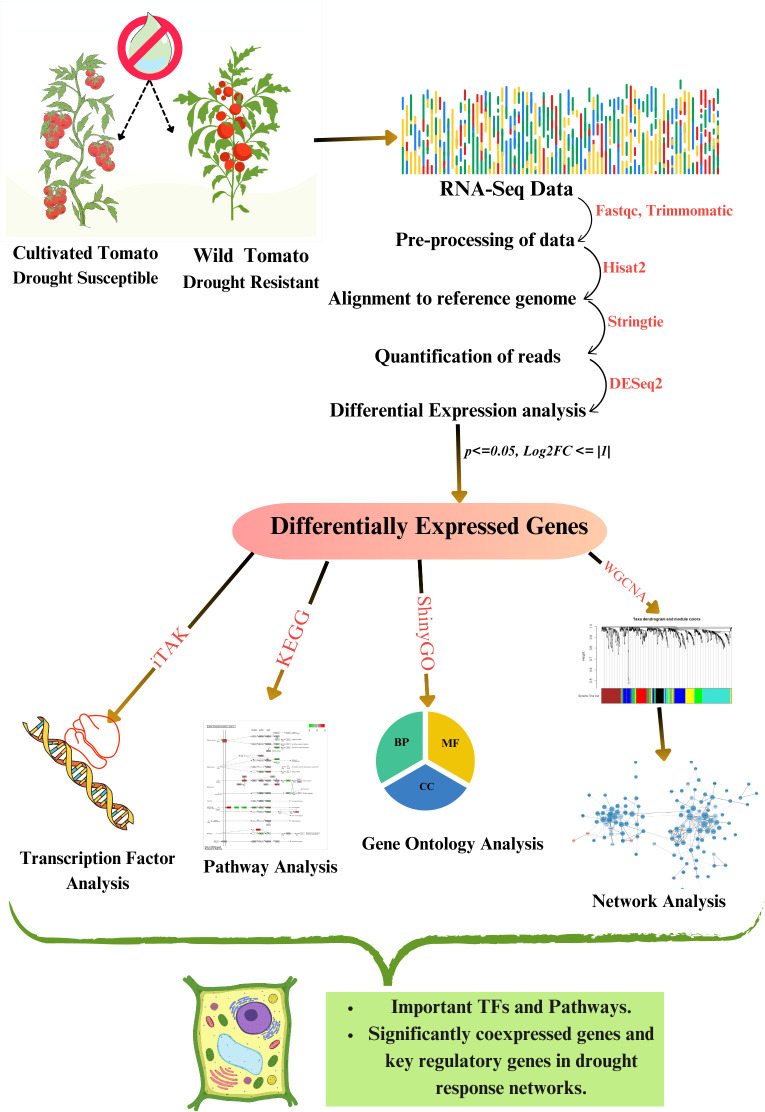
Methodology adopted in this study.

The leaf transcriptome dataset PRJNA800740 retrieved from the NCBI SRA database (Home - SRA - NCBI, 2024) consisted of drought-treated tolerant variety *Solanum pennellii* and susceptible variety *Solanum lycopersicum* respectively, obtained at three different time points of 2 weeks +5 days (2W+ 5D), 2 weeks +8 days (2W+ 8D), 2 weeks +11 days (2W+ 11D) of drought treatment in triplicates. The workflow of the current study is illustrated in **Figure 1**.

### 2.2 RNA-seq data pre-processing and assembly

The RNA-Seq data of both species were analyzed through the Galaxy suite (Galaxy, 2024), by using dataset fastq files as input in the fastQC tool (Andrews, 2010) for quality assessment of raw RNA-seq data. The quality-checked reads were mapped to the *Solanum lycopersicum* reference genome retrieved from the NCBI using HiSAT2 (Kim et al., 2019) and raw read counts of the mapped transcripts were obtained using the Stringtie tool (Shumate et al., 2022).

### 2.3 Differential gene expression analysis

Transcripts with a criteria of 5-10 counts per million (CPM) in at least three samples obtained from the Stringtie tool were selected for differential expression analysis, which was carried out using DESeq2 (Love et al., 2014). Pairwise comparisons between time points (e.g., day 5 vs. day 8 vs day 11) were performed to identify DEGs at key stages of drought stress, given the focus of the study on significant expression changes between early and late drought phases. With only three time points (days 5, 8, and 11), this approach was preferred over time-series models due to limited temporal resolution. Significant DEGs were identified using DESeq2 with an FDR-adjusted p-value (padj) < 0.05 and |log2 fold change| > 1. The Benjamini-Hochberg procedure was applied to correct for multiple testing and control the false discovery rate. The samples were named Wild Type_Day5 (WT_D5), Wild Type_Day8 (WT_D8), Wild Type_Day11 (WT_D11), Cultivated Type_Day5 (CT_D5), Cultivated Type_Day8 (CT_D8) and Cultivated Type_Day11 (CT_D11).

### 2.4 Pathway analysis

Differentially expressed genes (DEGs) identified from RNA-sequencing data were subjected to pathway enrichment analysis to uncover associated biological processes and signaling pathways. Functional annotation and pathway enrichment were performed using ShinyGO 0.82 (Ge et al., 2020), based on the KEGG pathway database. For pathway mapping, DEGs were provided as Ensembl IDs and a pathway was considered significantly enriched if the adjusted p-value (FDR) was < 0.05. Enrichment scores, and gene counts were extracted for the top-ranked pathways.

### 2.5 TF prediction

The iTAK version 18.12 software (Zheng et al., 2016) was used to identify transcription factors among the differentially expressed genes. iTAK is a program to identify plant transcription factors (TFs), transcriptional regulators (TRs) and protein kinases (PKs) from protein or nucleotide sequences, and then classify individual TFs, TRs and PKs into different gene families.

### 2.6 Weighted gene co-expression network analysis

Weighted gene co-expression network analysis (WGCNA) (Langfelder and Horvath, 2008) was performed on variance stabilizing transformation (vst) normalized genes to construct a co-expression network and further identified significant co-expressed genes at different drought stages. The vst normalized read count matrix obtained from Stringtie and DESeq2 pipeline was used for the analysis. The optimal parameters for constructing a weighted adjacency matrix, essential for capturing the intricate relationships among genes, were chosen using picksoftthreshold. The resulting matrix was transformed into a topology overlapping matrix (TOM) and later to dissTOM (1-TOM) allowing a comprehensive assessment of network connectivity. Later unsupervised hierarchical clustering was performed to identify gene modules exhibiting similar expression patterns, with further refinement achieved through the DynamicTreecut algorithm. The relationship between the gene modules and drought stages, such as 2W_5D, 2W_8D, and 2W_11D days was derived using a module-trait association study and is illustrated as a heatmap. By focusing on the module with the strongest positive correlation to drought phase traits, the key regulatory module was identified. This module was subjected to additional analysis to uncover biological significance in governing drought responses in both species.

The genes of the selected module were then used to create the protein-protein interaction network using the Stringapp plugin (Doncheva et al., 2019) in the Cytoscape tool (Shannon et al., 2003). A confidence score of >= 0.7 (high confidence) was applied and the resulting network was analyzed through Cytoscape plugins like network analyzer. Enriched pathways were identified using the string enrichment plugin in Cytoscape and significant hubs were found using the degree centrality of Network analyzer plugin (Chin et al., 2014).The genes in the blue module were also subjected to GO analysis and pathway analysis using the STRINGdb.

## 3 Results and discussion

### 3.1 RNA seq analysis

The 36 RNA-seq samples of WT and CT varieties of *Solanum* obtained from the NCBI SRA database underwent a FASTQC quality check which reported no adapter contamination and was further mapped to the reference genome *S. lycopersicum*. The average mapping ratio for the wild type was ~82% and that of the cultivated one was ~96%.

### 3.2 Differential expression analysis of two contrasting *Solanum* genotypes across all time points

A total of 2175 DEGs were obtained at three drought stress time points for two varieties, including 1459 in CT and 716 in WT based on an FDR-adjusted p-value (padj) < 0.05 and |log2FC| > 1(**Supplementary Table 1**). The number of upregulated genes was higher in most of the time points (WT_D5, WT_D11, CT_D8, and CT_D11). It was noticed that DEGs in WT was 8 at day 5 which was increased to 663 at day 11. Meanwhile, the number of DEGs in CT increased from 76 on day 5 to 1233 on day 11.

Comparing the DEGs at day 5 on both species, 1 gene was found to be in common with 75 DEGs specific to CT and 7 DEGs specific to WT. On day 8, the shared genes were increased to 21 with 129 CT-specific and 24 WT-specific genes. Drought response on day 11 shared 372 genes between both species with 861 exclusively expressed in WT and 291 expressed in CT (**Supplementary Table 1**).

In conclusion, the analysis of differential gene expression under drought stress across three time points revealed significant temporal and varietal differences in the transcriptional responses of the two varieties, CT and WT. Comparative analysis highlighted overlapping and distinct gene expression patterns, with shared DEGs between the varieties increasing over time. Despite the overlap, variety-specific DEGs were substantial, emphasizing the distinct mechanisms employed by CT and WT in drought adaptation. These findings provide insights into the temporal dynamics and variety-specific strategies in drought response. Additionally, the genes which were differentially expressed in all three days of drought were identified and analysed in the subsequent section.

#### 3.2.1 Genes present in all three times points and their expression pattern

**Figure 2:**
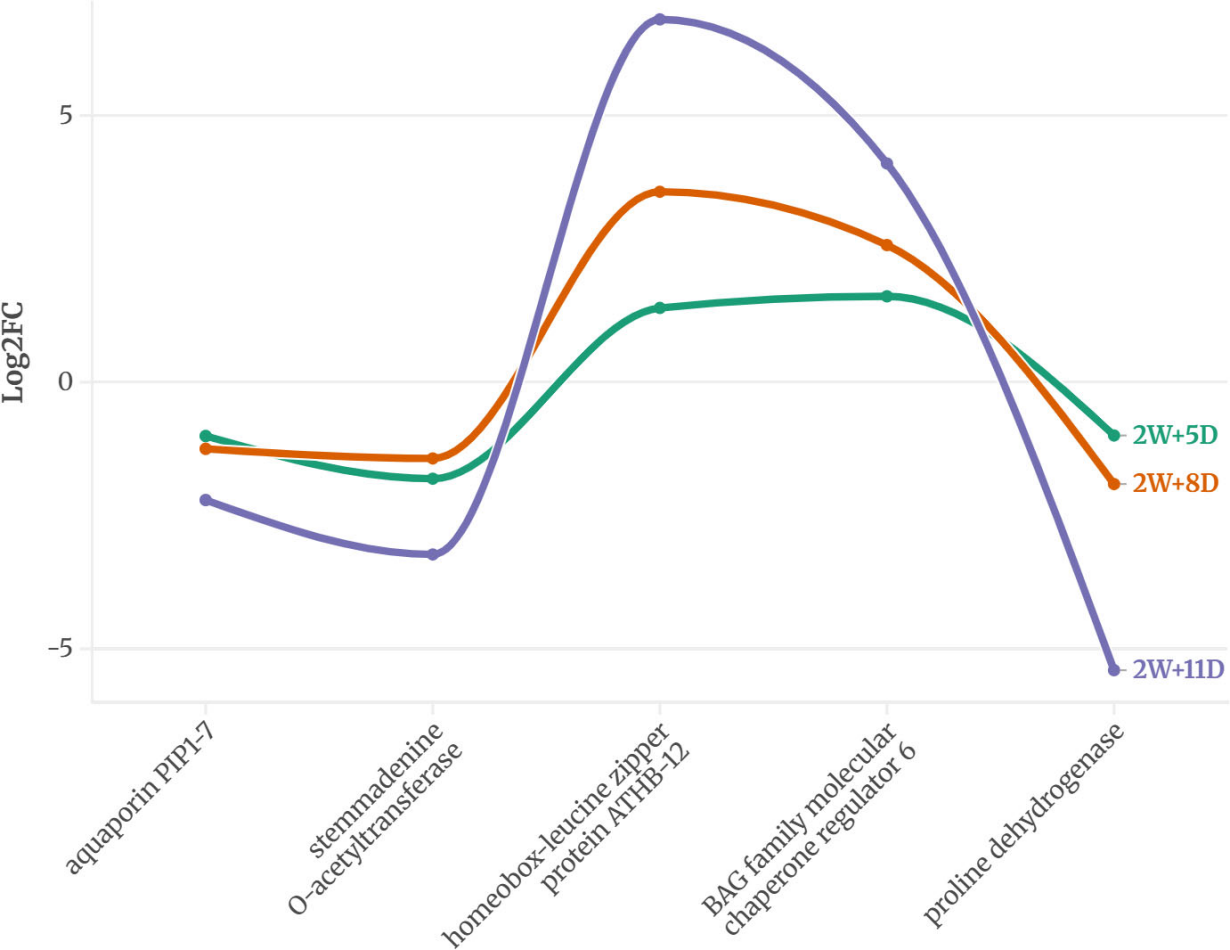
Common DEGs expressed on all three time points of WT identified using an FDR-adjusted p-value (padj) < 0.05 and |log2FC| > 1.

**Figure 3:**
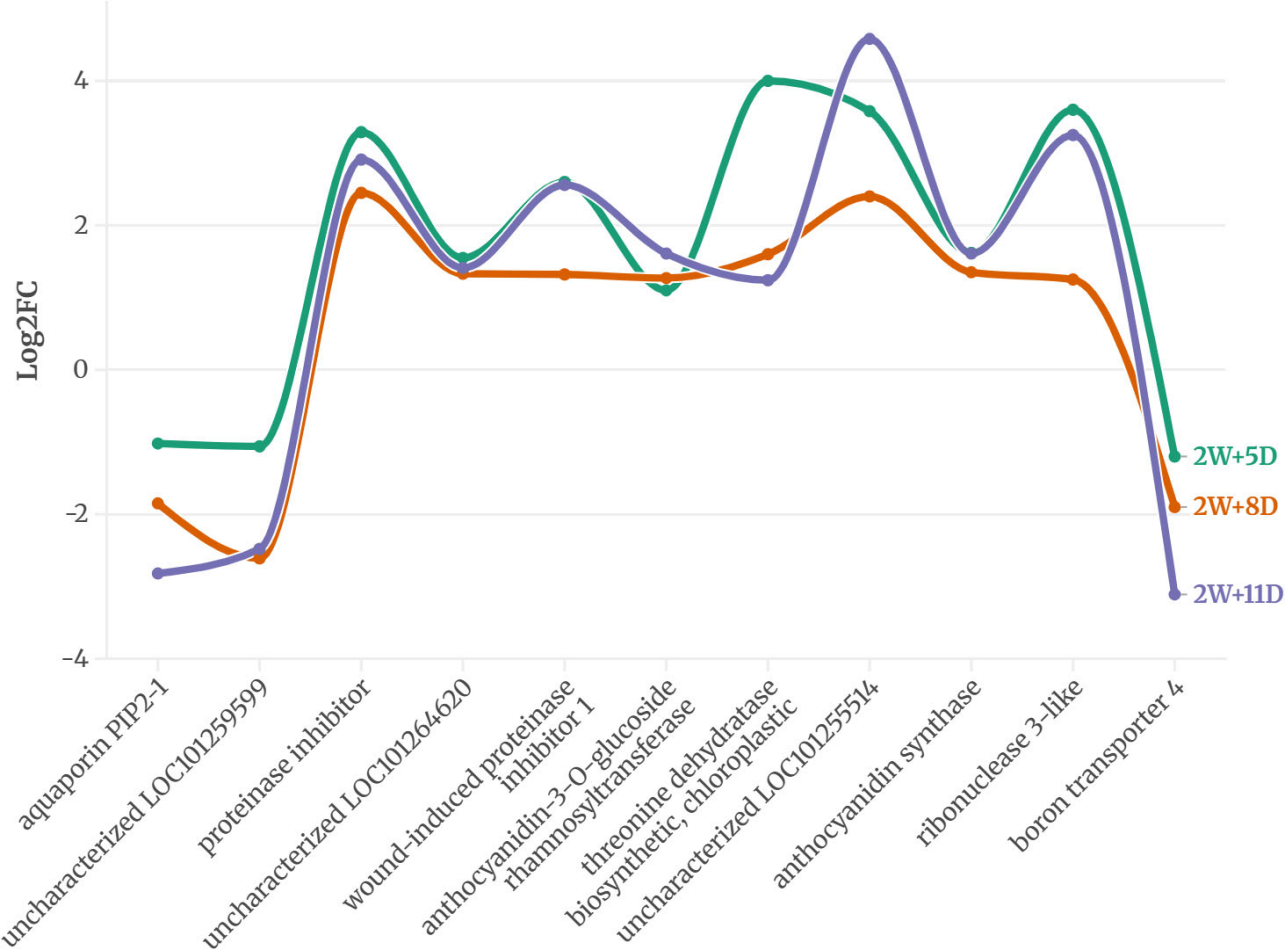
Common DEGs expressed on all three-time points of CT identified using an FDR-adjusted p-value (padj) < 0.05 and |log2FC| > 1.

Across all time points, 5 genes in WT and 11 in CT were consistently expressed, suggesting a potential role in drought response. The expression pattern of these proteins is provided in **Figures 2** and **3**.

In the drought-tolerant *S. pennellii*, five proteins were consistently expressed across all drought stages (**Figure 2**). The down regulated proteins were aquaporin PIP1-7, stemmadenine O-acetyltransferase, and proline dehydrogenase, with log2 fold changes ranging from -1.0 to -5.4. Proline dehydrogenase showed the most significant downregulation (-5.4 at 2W+11D), suggesting reduced proline catabolism, which may contribute to osmoprotectant accumulation (Hayat et al., 2012). Homeobox-leucine zipper protein ATHB-12 and BAG family molecular chaperone regulator 6 exhibited strong upregulation, with log2 fold changes peaking at 6.8 and 4.1, respectively, at 2W+11D. These proteins likely play roles in transcriptional regulation (Olsson et al., 2004) and protein homeostasis (Echevarría-Zomeño et al., 2016) under drought stress. The upregulated proteins showed a progressive increase in expression over time, particularly ATHB-12, which increased from 1.39 (2W+5D) to 6.8 (2W+11D), indicating a sustained and amplified stress response in the tolerant variety.

On the other hand, in the drought-sensitive *S. lycopersicum*, eleven proteins were consistently expressed across all three drought stages (**Figure 3**). Downregulated proteins included aquaporin PIP2-1, uncharacterized LOC101259599, and boron transporter 4, with log2 fold changes ranging from -1.02 to -3.11 by 2W+11D, indicating a strong suppression of water and nutrient transport mechanisms under prolonged drought. Proteinase inhibitor, uncharacterized LOC101255514, ribonuclease 3-like, wound-induced proteinase inhibitor 1, anthocyanidin synthase, anthocyanidin-3-O-glucoside rhamnosyltransferase, and threonine dehydratase biosynthetic (chloroplastic) showed positive log2 fold changes, ranging from 1.1 to 4.58. The highest upregulation was observed for uncharacterized LOC101255514 (4.58 at 2W+11D) and threonine dehydratase (4.0 at 2W+5D), suggesting roles in stress response, secondary metabolite production, and amino acid metabolism. Most upregulated proteins maintained or increased expression by 2W+11D, except for threonine dehydratase, which decreased from 4.0 (2W+5D) to 1.24 (2W+11D), indicating a possible early stress response that diminishes over time.

The differential protein expression profiles between CT and WT highlight distinct strategies for coping with drought stress. In the drought-sensitive CT, the downregulation of aquaporin PIP2-1 and boron transporter 4 suggests a reduction in water and nutrient uptake (Conti et al., 2022), potentially exacerbating drought susceptibility. Drought can lead to a higher concentration of boron in the soil solution due to reduced water availability. This can make plants more susceptible to boron toxicity, which can manifest as leaf necrosis and stunted root growth (Aftab et al., 2022). Aquaporins facilitate water transport across membranes, and their suppression may limit water movement, leading to cellular dehydration under prolonged stress (Chaumont and Tyerman, 2014). Conversely, the upregulation of proteinase inhibitors, ribonuclease 3-like, and anthocyanidin-related proteins indicates an activation of defense mechanisms, including protection against oxidative stress, drought (D’Ippólito et al., 2021) and pathogen attack (Zhang et al., 2024), which are often exacerbated during drought.

The high expression of threonine dehydratase early in drought (2W+5D) may support isoleucine biosynthesis, providing precursors for stress-related metabolites (Joshi et al., 2010), though its decline by 2W+11D suggests a limited capacity to sustain this response. In contrast, the drought-tolerant WT exhibits a more robust adaptive response. The downregulation of proline dehydrogenase likely contributes to proline accumulation (Junaid et al., 2023), a well-known osmoprotectant that stabilizes cellular structures under water deficit (Yamada et al., 2005) (Wang et al., 2023). The strong upregulation of homeobox-leucine zipper protein ATHB-12, a transcription factor (Li Y. et al., 2022), suggests enhanced regulation of drought-responsive genes, potentially orchestrating a coordinated stress response. Many studies have reported the role of homeobox-leucine zipper protein ATHB-12 during plant abiotic stress (Ariel et al., 2010) (Shen et al., 2019) (Deng et al., 2002) Similarly, the BAG family molecular chaperone regulator 6, involved in protein folding and stress tolerance, likely supports cellular homeostasis under prolonged drought (Arif et al., 2024). The progressive increase in expression of these proteins over time indicates a sustained and adaptive response, contrasting with the more variable expression in CT.

These observations suggest that WT employs a combination of osmoprotection, transcriptional regulation, and protein homeostasis to tolerate drought, whereas CT relies on stress defense mechanisms that may be less effective under prolonged drought. The proteins identified in WT, particularly proline dehydrogenase, ATHB-12, and BAG6, are promising candidates for studying drought tolerance in tomato varieties.

#### 3.2.2 Top up/downregulated genes in all three time points

**Figure 4:**
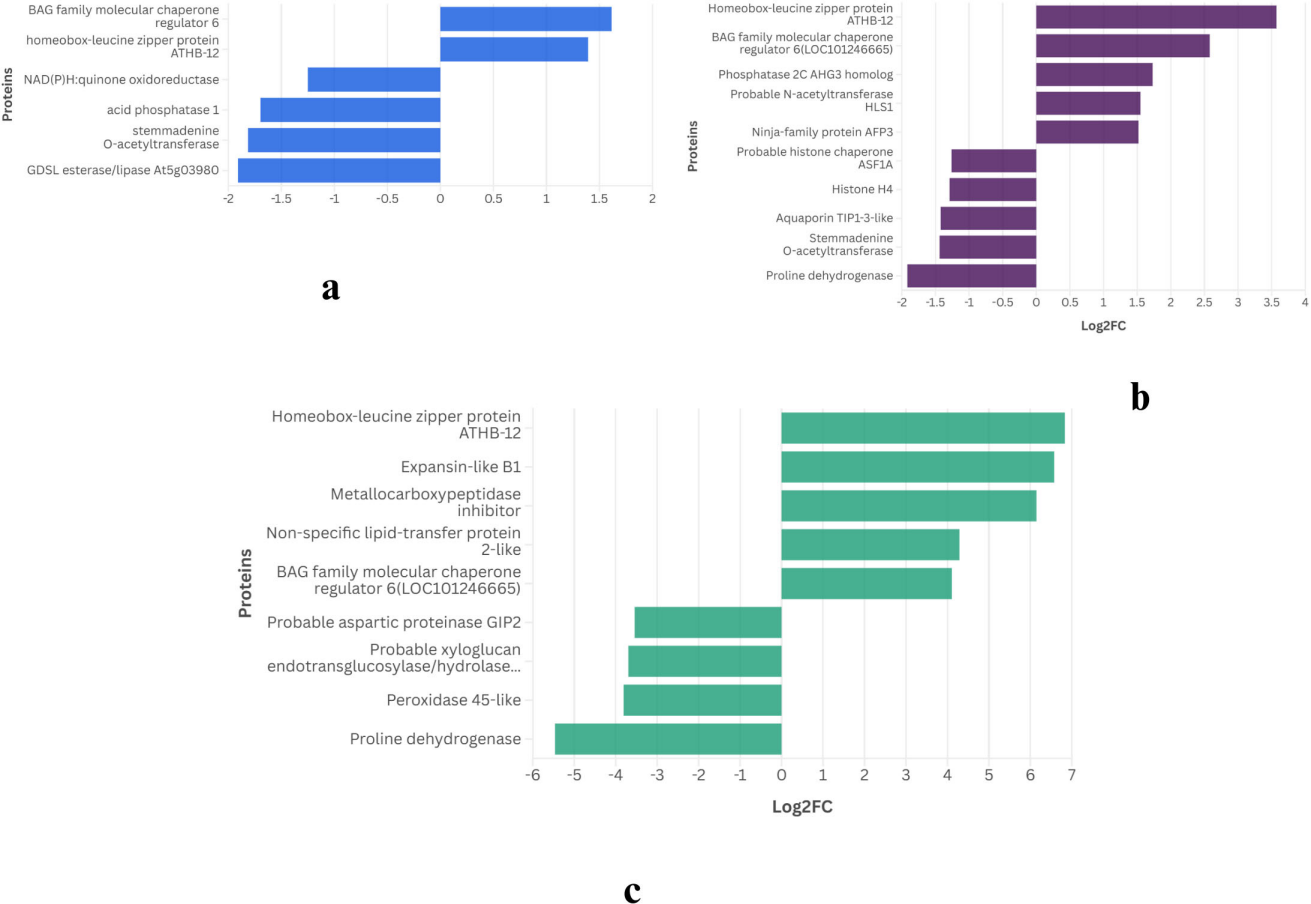
Top up/downregulated in day **(a)** 2W_5D **(b)** 2W_8D **(c)** 2W_11D of WT.

**Figure 5:**
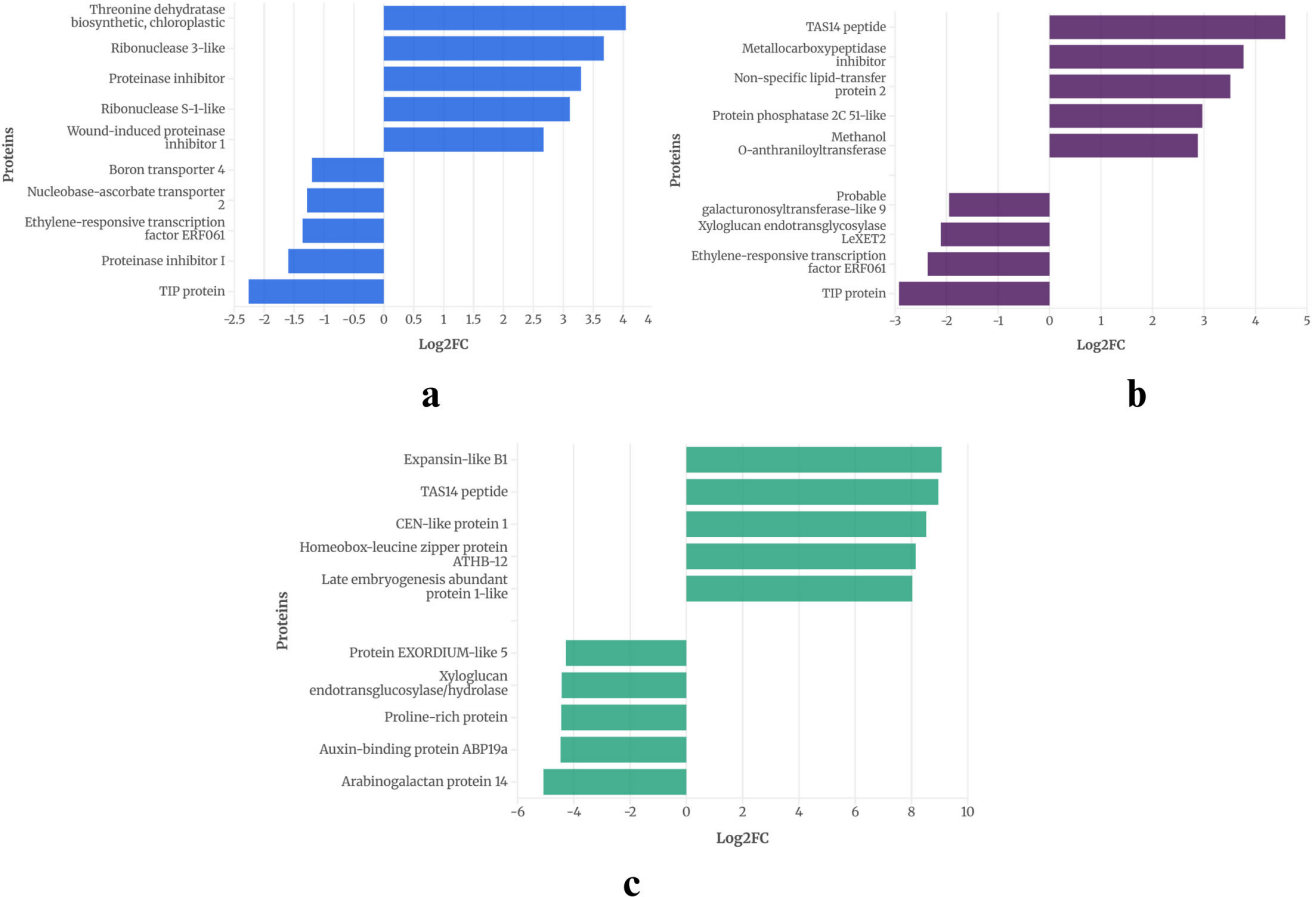
Top up/downregulated in day **(a)** 2W_5D **(b)** 2W_8D **(c)** 2W_11D of CT.

To illustrate the overall response of DEGs, the top five upregulated and downregulated genes from each of the three drought treatment days were analyzed. This analysis revealed distinct patterns of pathway enrichment, as shown in **Figures 4** and **5**.

At 2W + 5D of drought stress in WT, (**Figure 4a**), the most significantly upregulated genes included BAG family molecular chaperone regulator 6 (Log2FC ≈ 1.6) and homeobox-leucine zipper protein ATHB-12 (Log2FC ≈ 1.4). Studies like 9617808 report similar observations of homeobox-leucine zipper protein ATHB-12 in *Arabidopsis* and (Arif et al., 2021) reports AtBAG6, a close homolog BAG2, to be upregulated under drought and ABA, enhancing drought survival. Notable downregulated genes were NAD(P)H:quinone oxidoreductase, acid phosphatase 1, both with Log2FC values around -1.5 and O-acetyltransferase and GDSL esterase/lipase At5g03980 were downregulated, with Log2FC values of approximately -2, respectively. By 2W + 8D (**Figure 4b**), the gene expression profile shifted. Homeobox-leucine zipper protein ATHB-12 remained highly upregulated (Log2FC ≈ 3.5), alongside BAG family molecular chaperone regulator 6 (Log2FC ≈ 2.5). Other upregulated genes included phosphatase 2C AHG3 homolog (Log2FC ≈ 1.5), probable N-acetyltransferase HLS1 (Log2FC ≈ 1.5), and ninja-family protein AFP3 (Log2FC ≈ 1.5). Genes like probable histone chaperone ASF1A, histone H4, and aquaporin TIP1-3-like and stemmadenine O-acetyltransferase showed moderate downregulation (Log2FC ≈ -1 to -1.5). Proline dehydrogenase was downregulated (Log2FC ≈ -2). At 2W + 11D (**Figure 4C**), the expression of homeobox-leucine zipper protein ATHB-12 continued to dominate (Log2FC ≈ 7), followed by expansin-like B1 (Log2FC ≈ 6.5) and metallocarboxypeptidase inhibitor (Log2FC ≈ 6). Similar upregulation of expansin-like B1 is also reported by (Gonçalves et al., 2019) in sweet oranges during drought stress. Non-specific lipid-transfer protein 2-like and BAG family molecular chaperone regulator 6 were also upregulated, with Log2FC values of approximately 4. Genes such as probable aspartic proteinase G1P2, probable xyloglucan endotransglucosylase/hydrolase and peroxidase 45-like showed downregulation (Log2FC ≈ -3.5 to -4). Proline dehydrogenases were significantly downregulated, with Log2FC values of approximately -5.5. Proline dehydrogenase genes as mentioned in the earlier section are consistently reported to be downregulated under drought, which leads to proline accumulation, a known osmoprotectant in plants.

The progressive increase in ATHB-12 expression across all stages (Log2FC from 1.4 to 7) highlights its pivotal role in drought stress adaptation, potentially acting as a master regulator of stress-responsive gene networks. The consistent upregulation of BAG6 further emphasizes the importance of protein homeostasis in mitigating drought-induced cellular damage. The downregulation of metabolic and growth-related genes, particularly proline dehydrogenase, underscores a strategic shift toward osmoprotection and resource conservation as drought severity increases. These findings align with previous studies on drought stress in Arabidopsis, where transcriptional reprogramming prioritizes stress tolerance over growth (Nakashima et al., 2025).

On the other hand, in CT, At 2W + 5D of drought stress, the most significantly upregulated genes included threonine dehydratase (Log2FC ≈ 4) and ribonuclease 3-like (Log2FC ≈ 3.5) (**Figure 5A**). Threonine dehydratase, also known as threonine deaminase, plays a crucial role in amino acid metabolism, specifically catalyzing the conversion of threonine to α-ketobutyrate and ammonia. This reaction is the first step in the isoleucine biosynthesis pathway. Although (Ziegler et al., 2025) reports a downregulation of threonine dehydratase gene in *Ribes nigrum* L during drought stress, its specific role in Solanum drought response is less explored. RNase 3-like belongs to the RNase III family, which is involved in processing double-stranded RNA (dsRNA) and regulating RNA stability, including mRNA, rRNA, and small RNAs (Comella et al., 2008). The role of ribonuclease 3-like is not extensively detailed in the literature but can likely contribute to drought stress by regulating RNA turnover and processing small RNAs to modulate stress-responsive gene networks. Other upregulated genes were proteinase inhibitor (Log2FC ≈ 3.5), ribonuclease S-1-like (Log2FC ≈ 3.1), and wound-induced proteinase inhibitor 1 (Log2FC ≈ 2.6). Proteinase inhibitors are small proteins that inhibit the activity of proteases (e.g., serine, cysteine, or metalloproteases), preventing excessive protein degradation during stress. The upregulation of proteinase inhibitor (Log2FC ≈ 3.5) and wound-induced proteinase inhibitor 1 in CT at 2W + 5D suggests a key role in stabilizing critical proteins, such as enzymes or structural components, to maintain cellular function (Huang et al., 2007). Interestingly, while certain proteinase inhibitor (PI) genes such as wound-induced proteinase inhibitor 1 were markedly upregulated under drought stress, others, including proteinase inhibitor I, showed moderate downregulation. This apparent contrast likely reflects the functional diversification within the proteinase inhibitor gene family. Proteinase inhibitors are a broad group encompassing multiple isoforms and families (e.g., type I, type II, wound-inducible, and ABA-responsive), each of which may be differentially regulated depending on the type, duration, and intensity of stress. The upregulation of wound- or ABA-inducible proteinase inhibitor under drought suggests a protective role in limiting stress-induced proteolysis and maintaining proteostasis. Conversely, the downregulation of proteinase inhibitor I may facilitate selective protease activity necessary for stress adaptation or signal transduction. Similar divergent expression patterns within the proteinase inhibitor gene family have been reported previously under abiotic stress conditions, including drought and salinity (Pautot et al., 1991) underscoring the finely tuned regulatory mechanisms that balance protein degradation and protection during stress responses. showed up-regulation of proteinase inhibitor, potato inhibitor I in drought-tolerant Sorghum variety was reported by (Goche et al., 2020).

Genes such as boron transporter 4, nucleobase-ascorbate transporter 2, ethylene-responsive transcription factor ERF061, proteinase inhibitor I showed moderate downregulation (Log2FC ≈ -1 to -1.5). TIP protein was the highest downregulated (Log2FC ≈ -2.3). These observations were consistent with previous reports where similar drought-responsive downregulation of TIPs was documented in barley (Kurowska et al., 2019) and common bean (Zupin et al., 2017), while decreased protease inhibitor expression in sensitive cereal genotypes has been reviewed (Moloi and Ngara, 2023). Additionally, drought-driven alterations in boron transporter expression were reported in *Arabidopsis* (Lv et al., 2017).

By 2W + 8D (**Figure 5B**), TAS14 peptide was the most upregulated (Log2FC ≈ 4.5), similar to the observations made by (Muñoz-Mayor et al., 2012). This was followed by metallo carboxypeptidase inhibitor (Log2FC ≈ 3.8) and non-specific lipid-transfer protein 2 (Log2FC ≈ 3.5) (Fernández et al., 2013). reviews plant metallocarboxypeptidase inhibitors as stress−responsive protease regulators and are known to increase under stress. Many drought studies in plants report upregulation of non-specific lipid-transfer protein 2 as under water deficit (Morales-Quintana et al., 2024) (Pan et al., 2016). This upregulation is thought to play a role in protecting plant cells and tissues from the damaging effects of water scarcity, specifically by influencing membrane stability and potentially aiding in the synthesis of protective compounds like cuticular wax.

Protein phosphatase 2C (PP2C) 51-like and methanol O-anthraniloyltransferase were also upregulated (Log2FC ≈ 3). Genome-wide analyses in tomato have identified PP2C family members as strongly responsive to drought stress, showing both elevated expression levels and enzyme activity under water deficit conditions (Sakib et al., 2024). While specific studies on anthraniloyltransferases are limited, transferase enzymes involved in secondary metabolism are commonly noted to increase under drought stress (Dixon et al., 2010).

Genes like probable galacturonosyltransferase-like 9, xyloglucan endotransglucosylase LexE12 and ethylene-responsive transcription factor ERF061 showed moderate downregulation (Log2FC ≈ -1 to -2.5). Similar to our observation, cell−wall biosynthesis enzymes such as galacturonosyltransferases are frequently shown to decrease in expression during drought, consistent with reduced growth and remodeling of the cell wall under stress in studies like (Veronico et al., 2022). XTH proteins that modify cell-wall structure were reported to have an expression dropping in water-stressed tomato tissues. Ethylene-responsive factors like ERF061 are often upregulated in drought-tolerant varieties, reflecting their role in growth and stress cross−talk (Liu et al., 2022).TIP protein remained downregulated (Log2FC ≈ -3).

At 2W + 11D (**Figure 5C**), expansin-like B1 was the most upregulated gene (Log2FC ≈ 8.5), followed by TAS14 peptide (Log2FC ≈ 8.4) and CEN-like protein 1 (Log2FC ≈ 8). Studies in potato and other crops show that cell wall modification protein, expansin−like B1 is strongly drought-induced (Ponce et al., 2022). TAS14 peptide expression is generally linked to improved drought tolerance by enhancing the plant’s ability to maintain osmotic balance (Muñoz-Mayor et al., 2012). The observed TAS14 upregulation in the drought-sensitive genotype may reflect an attempted but insufficient protective response, indicating that its induction alone is not sufficient to confer tolerance. Homeobox-leucine zipper protein ATHB-12 and late embryogenesis abundant protein 1-like were also significantly upregulated (Log2FC ≈ 8). LEA proteins accumulate strongly during late drought or desiccation phases to prevent protein aggregation (Goyal et al., 2005). Drought can interfere with flowering of a plant and therefore many plants hasten this process to shorten their life cycle under water scarcity. CEN-like protein 1 plays a role in regulating flowering time under drought stress (Wang et al., 2020). reported activation of this gene delaying flowering by interacting with other proteins like 14-3-3 proteins and OsFD1, forming a complex that suppresses the florigen. Genes such as protein EXORDIUM-like 5, xyloglucan endotransglucosylase/hydrolase (Log2FC ≈ 2), proline-rich protein, and auxin-binding protein ABP19a showed significant downregulation (Log2FC ≈ -4) along with arabinogalactan protein 14 was downregulated (Log2FC ≈ -5).

These findings suggest that the CT genotype employs a unique combination of protease inhibition, osmotic protection, and ABA signaling to cope with drought stress, differing from the focus on chaperone activity and proline accumulation by WT. The progressive upregulation of protective genes like TAS14 peptide and metallocarboxypeptidase inhibitor across stages highlights the emphasis of CT genotype on cellular protection and protein stabilization, distinct from the WT’s reliance on BAG6 and proline metabolism. The shared upregulation of ATHB-12 at 2W + 11D in both genotypes suggests a convergent regulatory mechanism under severe drought, though the CT genotype exhibits a broader suite of highly upregulated genes (Log2FC ≈ 8–8.5) compared to WT. The consistent downregulation of cell wall modification and growth-related genes (e.g., xyloglucan endotransglucosylase/hydrolase, arabinogalactan protein 14) in CT mirrors WT patterns, indicating a conserved strategy to minimize energy expenditure.

Drought tolerance in WT likely stems from its early and sustained activation of osmoprotective (proline dehydrogenase downregulation) and protein stabilization (BAG6) mechanisms, coupled with a balanced ATHB-12-mediated stress response that preserves some growth potential. The intense upregulation of protective genes and stronger growth suppression in CT suggests a less efficient, potentially reactive strategy that may compromise long-term resilience.

### 3.3 Pathways enriched during all three time points of drought in WT and CT

**Figure 6:**
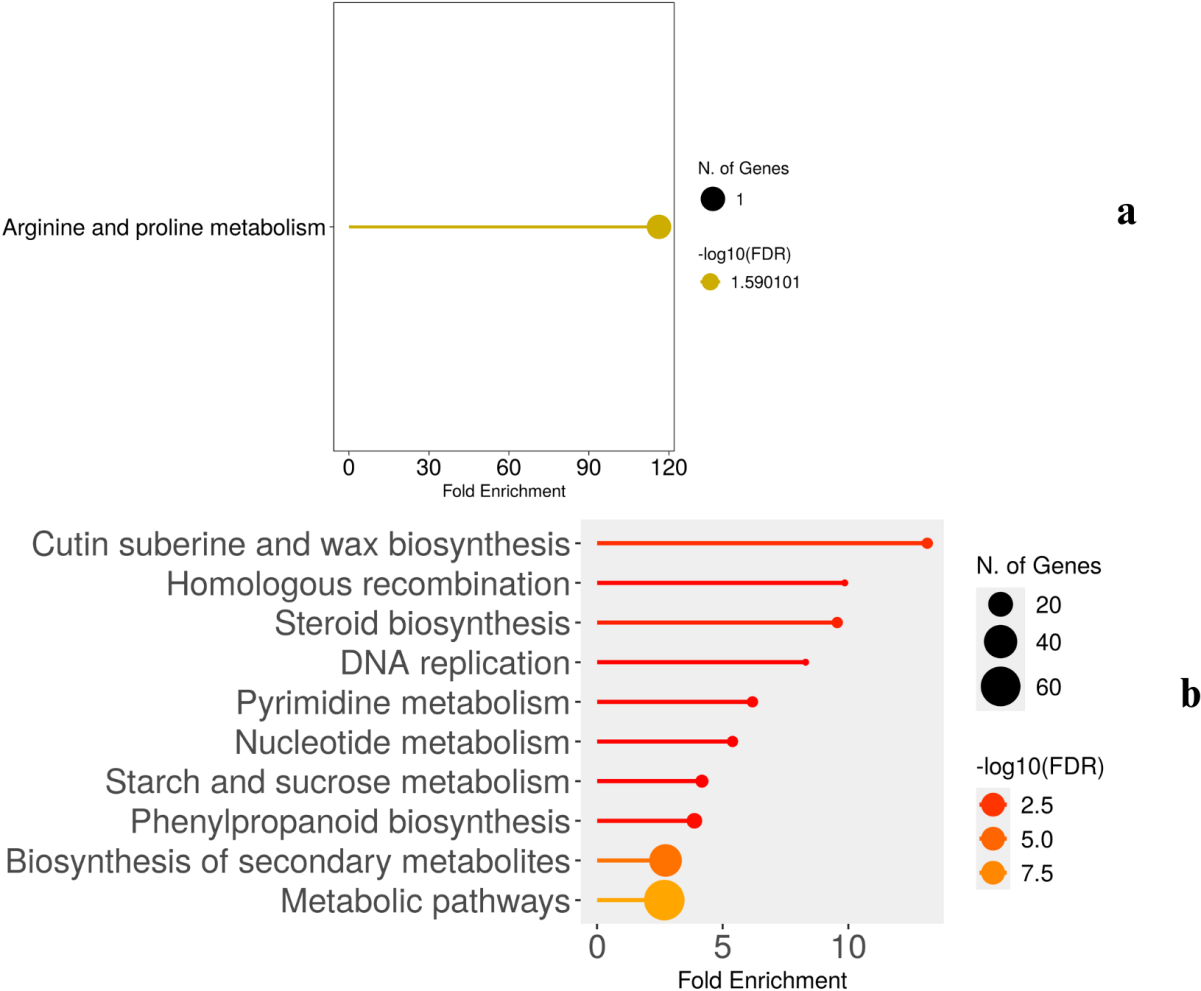
Major pathways enriched in WT in **(a)** 2W+5D and **(b)** 2W+11D days of drought.

**Figure 7:**
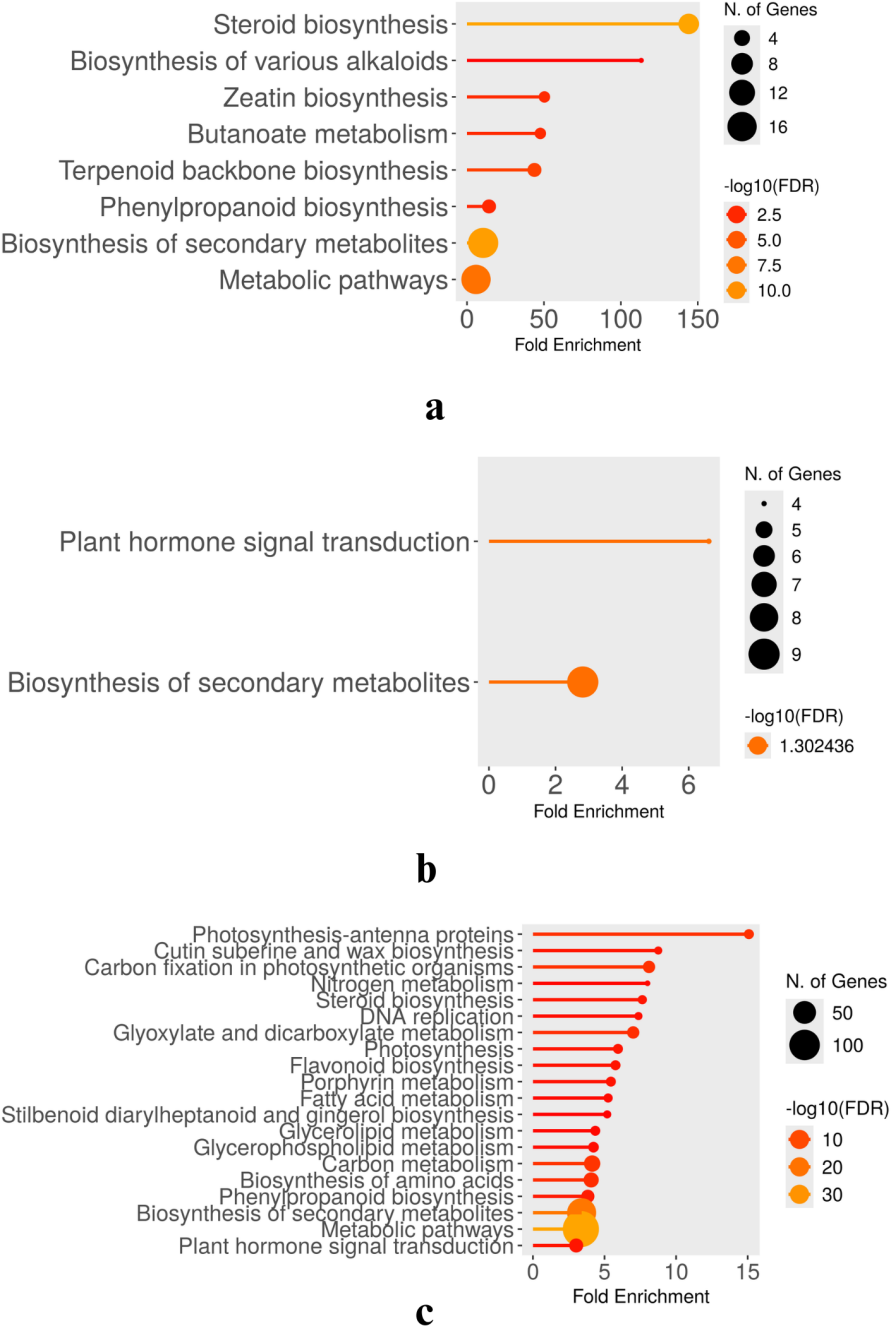
Major pathways enriched in CT in **(a)** 2W+5D **(b)** 2W+8D and **(c)** 2W+11D days of drought.

To gain an overview of the enriched drought response pathways on all three days of drought, the pathway analysis of differentially expressed genes were carried out using ShinyGo. The pathway enrichment profiles of wild-type (**Figure 6**) and cultivated (**Figure 7**) Solanum under drought stress reveal both similarities and distinct differences, reflecting their adaptive strategies. Detailed results are presented in **Supplementary Table 2**.

The pathway enrichment analysis of wild-type (WT) plants under drought stress at two time points (2W + 5D and 2W + 11D) revealed significant changes in metabolic pathways, as depicted in panels **Figures 6a, b**. Notably, no pathway enrichment was observed at 2W + 8D. At 2W + 5D of drought stress, only one pathway was significantly enriched: arginine and proline metabolism. This pathway showed a fold enrichment of approximately 120, with a single gene involved and a false discovery rate (FDR) of -log10(FDR) ≈ 1.59. By 2W + 11D, multiple pathways were significantly enriched. The most enriched pathway was cutin suberine and wax biosynthesis, with a fold enrichment of approximately 12 and 60 genes involved. This was followed by homologous recombination (fold enrichment ≈ 10, 40 genes, steroid biosynthesis (fold enrichment ≈ 8) and DNA replication (fold enrichment ≈ 7).

The pathway enrichment analysis highlights a temporal shift in the metabolic response of WT plants to drought stress. At 2W + 5D, the sole enrichment of arginine and proline metabolism suggests an early focus on osmotic adjustment. Proline accumulation as mentioned in previous sections is a well-documented drought response mechanism in plants, as it serves as an osmoprotectant to maintain cellular water balance under stress. The high fold enrichment (≈120) indicates that this pathway is strongly activated early in the drought response, even though only one gene was significantly involved. This also aligns with the earlier observation of proline dehydrogenase downregulation in WT plants (Log2FC ≈ -0.5 at 2W + 8D and -5 at 2W + 11D), supporting proline conservation for osmotic protection. The absence of pathway enrichment at 2W + 8D may indicate a transitional phase where the plant is shifting from early metabolic adjustments to more structural and protective mechanisms. This lack of enrichment could also reflect a temporary stabilization of gene expression as the plant adapts to ongoing stress, before a broader response is triggered by 2W + 11D. At 2W + 11D, the enrichment of multiple pathways reflects a more comprehensive drought response. The high enrichment of cutin suberine and wax biosynthesis (fold enrichment ≈ 12, 60 genes) indicates a focus on enhancing the plant’s cuticle layer, which reduces water loss through transpiration—a critical adaptation for prolonged drought (Chen et al., 2020) (Kosma et al., 2009). The enrichment of homologous recombination and DNA replication (fold enrichments ≈ 10 and 7, respectively) suggests that WT plants are actively repairing DNA damage caused by drought-induced oxidative stress and maintaining genome stability, which is essential for long-term survival (Banerjee and Roy, 2021). Steroid biosynthesis (fold enrichment ≈ 8) may contribute to the production of sterols, which stabilize cell membranes under stress (Kumar et al., 2018).

In comparison to the earlier gene expression data, the sustained downregulation of proline dehydrogenase in WT plants at 2W + 11D (Log2FC ≈ -5) aligns with the early enrichment of arginine and proline metabolism at 2W + 5D, reinforcing the importance of proline in WT drought tolerance. Furthermore, the upregulation of expansin-like B1 (Log2FC ≈ 5) and ATHB-12 (Log2FC ≈ 6) at 2W + 11D in WT plants likely contributes to the observed cutin suberine and wax biosynthesis enrichment, as these genes are involved in cell wall modification and transcriptional regulation of stress responses.

For CT, the pathway enrichment analysis of cultivated-type (CT) plants under drought stress at three time points (2W + 5D, 2W + 8D, and 2W + 11D) revealed distinct changes in metabolic pathways, as depicted in panels (a), (b), and (c) of **Figure 7**.

At 2W + 5D of drought stress, several pathways were significantly enriched. The most enriched pathway was steroid biosynthesis, with a fold enrichment of approximately 150 and 16 genes involved (-log10(FDR) ≈ 10.0). This was followed by biosynthesis of various alkaloids (fold enrichment ≈ 120, -log10(FDR) ≈ 7.5), zeatin biosynthesis (fold enrichment ≈ 50, -log10(FDR) ≈ 5.0), butanoate metabolism (fold enrichment ≈ 50, -log10(FDR) ≈ 5.0), and terpenoid backbone biosynthesis (fold enrichment ≈ 50, -log10(FDR) ≈ 5.0). Other pathways included phenylpropanoid biosynthesis (fold enrichment ≈ 40, -log10(FDR) ≈ 2.5), biosynthesis of secondary metabolites (fold enrichment ≈ 20, -log10(FDR) ≈ 2.5), and metabolic pathways (fold enrichment ≈ 10, -log10(FDR) ≈ 2.5).

At 2W + 8D, two pathways were significantly enriched. Plant hormone signal transduction showed a fold enrichment of approximately 6 (-log10(FDR) ≈ 1.30). Biosynthesis of secondary metabolites had a fold enrichment of approximately 4 (-log10(FDR) ≈ 1.30).

By 2W + 11D, multiple pathways were enriched, primarily related to photosynthesis and metabolism. Photosynthesis - antenna proteins and cutin suberine and wax biosynthesis were the most enriched, each with a fold enrichment of approximately 15 (-log10(FDR) ≈ 10). Other highly enriched pathways included carbon fixation in photosynthetic organisms (fold enrichment ≈ 14, -log10(FDR) ≈ 10), nitrogen metabolism (fold enrichment ≈ 13, -log10(FDR) ≈ 10), steroid biosynthesis (fold enrichment ≈ 12, -log10(FDR) ≈ 20), and DNA replication (fold enrichment ≈ 11, -log10(FDR) ≈ 10). Pathways such as glyoxylate and dicarboxylate metabolism, photosynthesis, porphyrin metabolism, fatty acid metabolism, stilbenoid diarylheptanoid and gingerol biosynthesis, glycerolipid metabolism, carbon metabolism, biosynthesis of amino acids, phenylpropanoid biosynthesis, biosynthesis of secondary metabolites, metabolic pathways, and plant hormone signal transduction showed fold enrichments ranging from 3 to 10 and -log10(FDR) values ranging from 10 to 30.

The pathway enrichment analysis of CT plants under drought stress reveals a distinct temporal response compared to WT plants, reflecting differences in their adaptive strategies. At 2W + 5D, the strong enrichment of steroid biosynthesis and biosynthesis of various alkaloids suggests an early focus on producing sterols and secondary metabolites, which may stabilize membranes and provide antioxidant protection under stress. Zeatin biosynthesis indicates a potential role for cytokinins in delaying senescence and maintaining cell division under drought. Butanoate metabolism and terpenoid backbone biosynthesis further support the production of secondary metabolites and energy intermediates, while phenylpropanoid biosynthesis points to the synthesis of lignins and flavonoids for cell wall reinforcement and ROS scavenging.

At 2W + 8D, the enrichment of plant hormone signal transduction aligns with the earlier gene expression data, where ethylene-responsive transcription factor ERF061 and protein phosphatase 2C 51-like were upregulated, indicating a role for ethylene and ABA signaling in the mid-stage drought response. The enrichment of biosynthesis of secondary metabolites continues the trend of producing protective compounds, though the limited number of enriched pathways at this stage suggests a more focused response compared to the broader metabolic reprogramming seen in WT plants.

By 2W + 11D, the enrichment of photosynthesis-related pathways such as photosynthesis - antenna proteins, carbon fixation in photosynthetic organisms, and photosynthesis indicates an attempt to maintain photosynthetic efficiency despite prolonged drought. This is surprising, as drought typically suppresses photosynthesis due to stomatal closure; the enrichment may reflect a compensatory mechanism in CT plants to sustain energy production. Cutin suberine and wax biosynthesis mirrors the WT response at this stage, suggesting a conserved strategy to reduce water loss through enhanced cuticle formation. The enrichment of steroid biosynthesis and DNA replication indicates continued membrane stabilization and genome maintenance, while nitrogen metabolism, glyoxylate and dicarboxylate metabolism, and carbon metabolism reflect metabolic adjustments to optimize resource use under stress. The presence of plant hormone signal transduction and biosynthesis of secondary metabolites further supports a role for hormonal regulation and protective metabolite production.

In comparison to the gene expression data, the upregulation of TAS14 peptide and expansin-like B1 at 2W + 11D in CT plants aligns with the enrichment of cutin suberine and wax biosynthesis, as these genes contribute to osmotic protection and cell wall modification. However, unlike WT plants, CT plants do not show significant enrichment of arginine and proline metabolism at any stage, consistent with the lack of proline dehydrogenase regulation observed in earlier gene expression data, which may limit their osmotic adjustment capacity.

Overall, CT plants exhibit a drought response focused on secondary metabolite production and hormonal signaling in the early and mid-stages, transitioning to a photosynthesis-centric strategy by 2W + 11D. While this response includes some protective mechanisms like cuticle enhancement, the lack of proline metabolism enrichment and the heavy reliance on maintaining photosynthesis under severe stress may indicate a less robust adaptation compared to WT plants, as discussed previously.

### 3.4 TFs genes in response to drought stress in both varieties of *Solanum*

**Figure 8:**
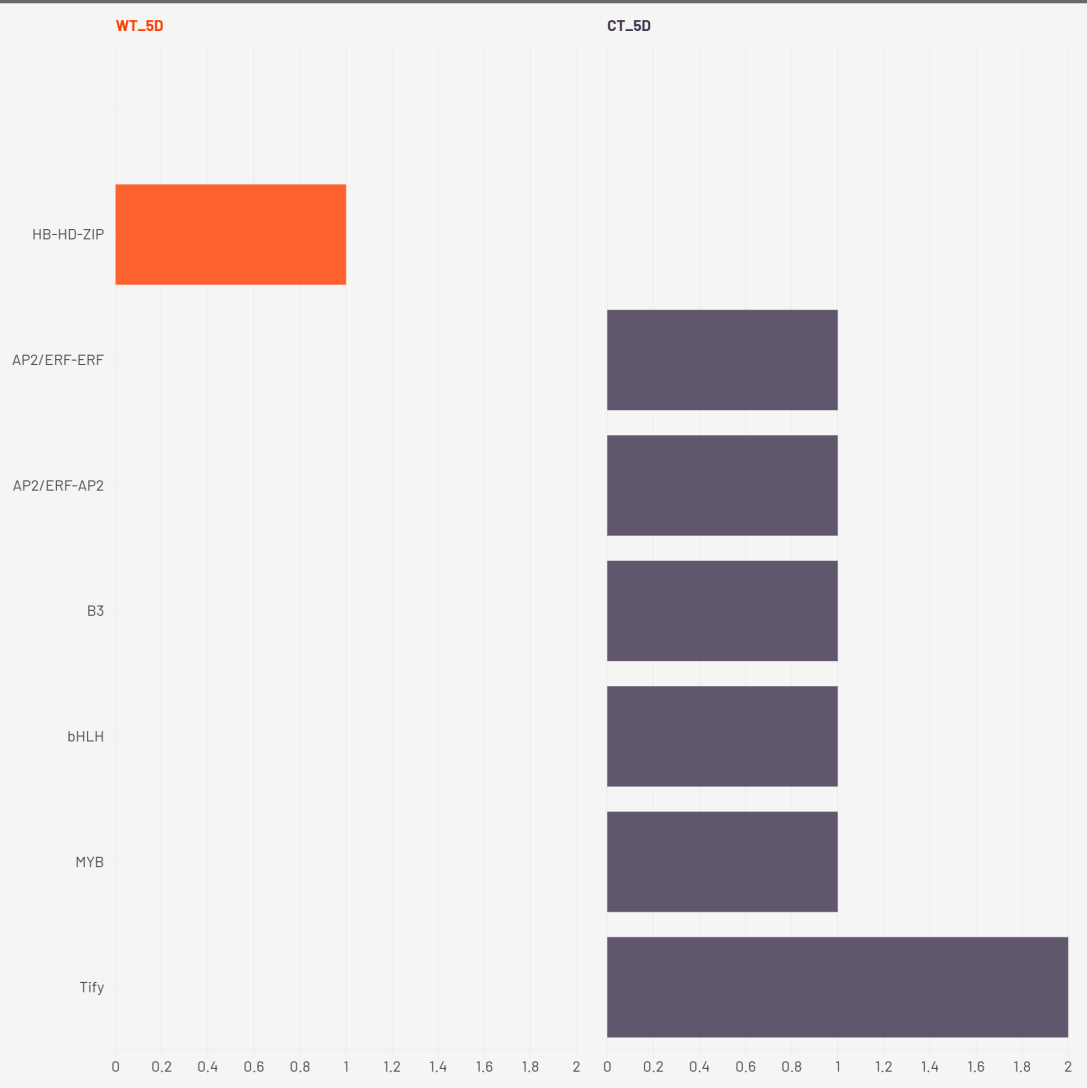
Enriched transcription factors in both CT and WT at 2W+5D.

**Figure 9:**
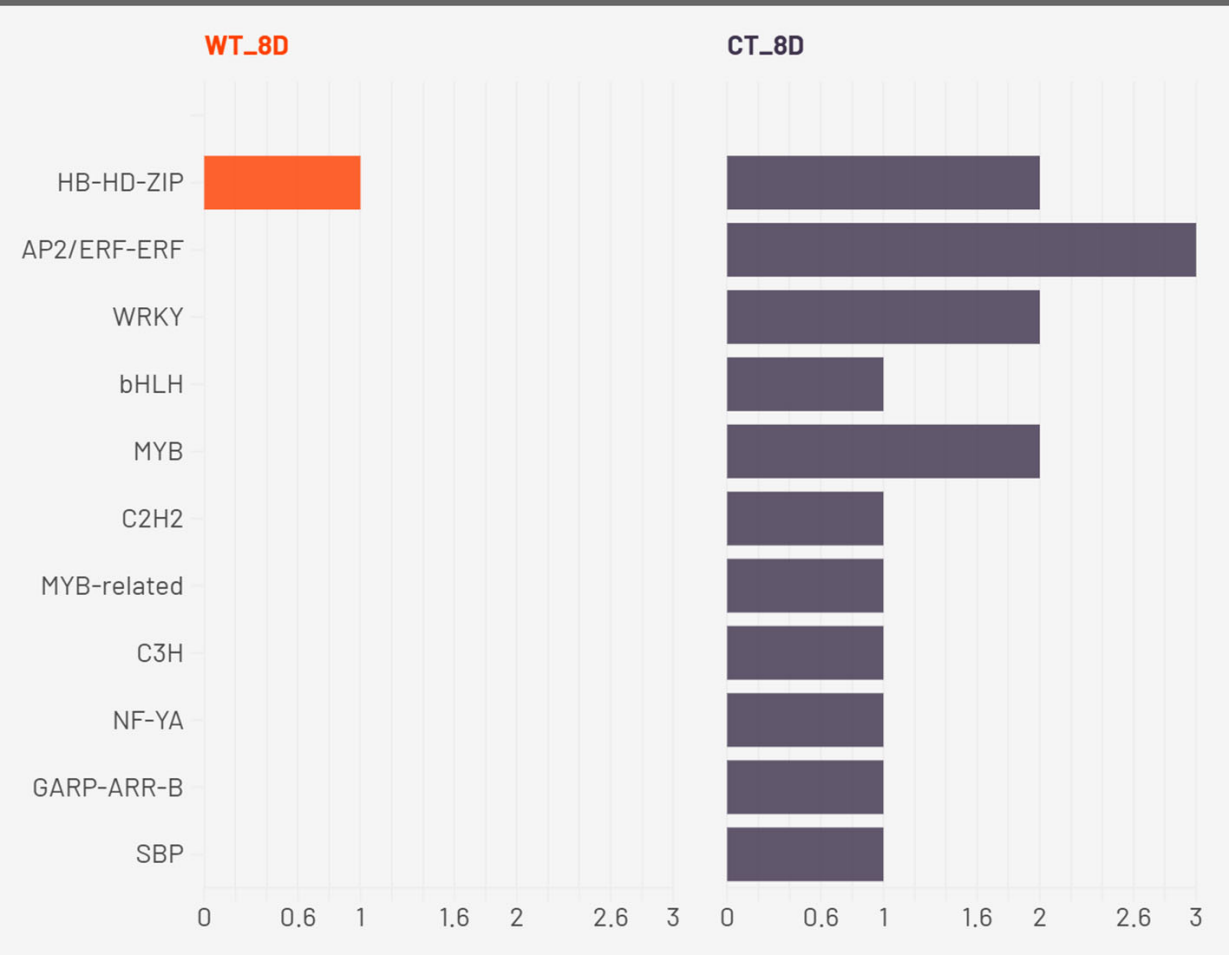
Enriched transcription factors in both CT and WT at 2W+8D.

**Figure 10:**
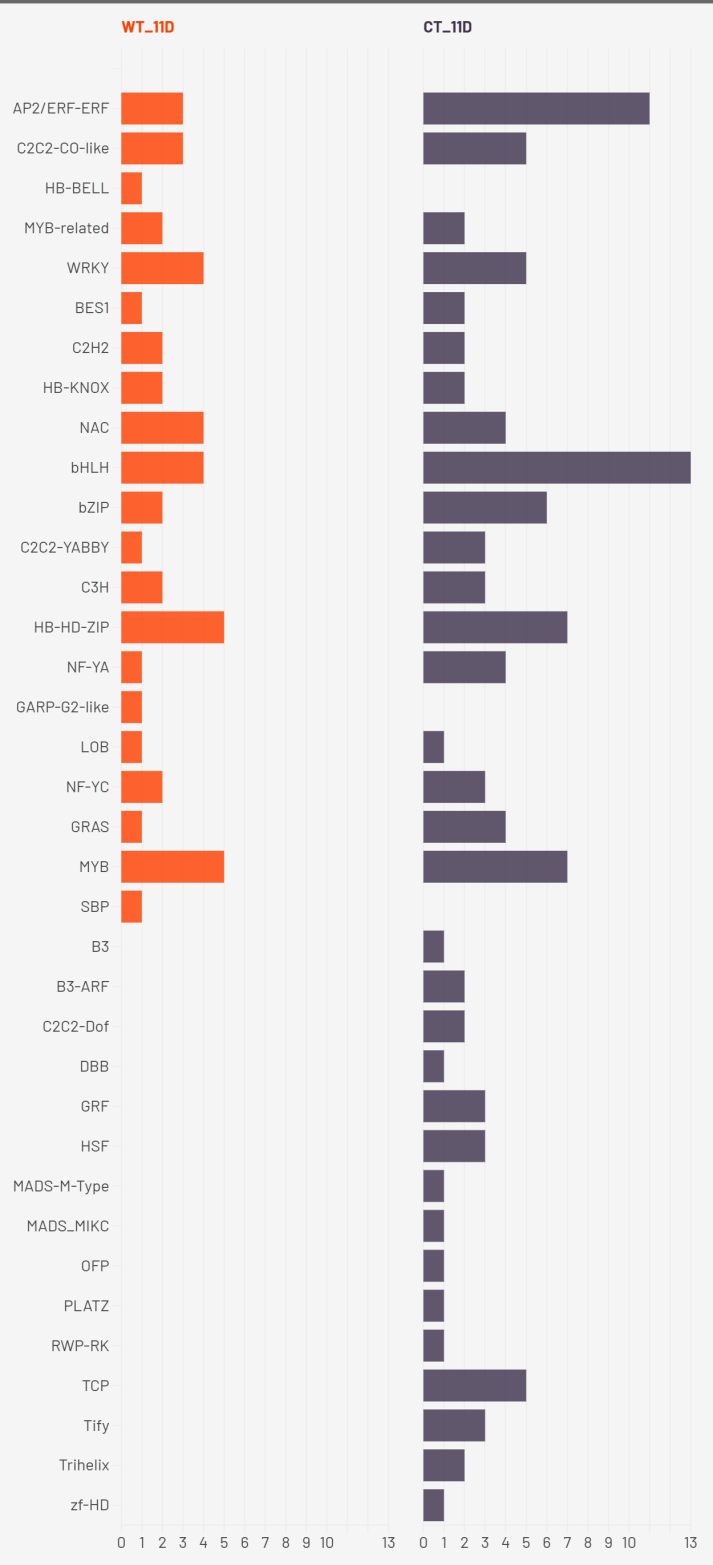
Enriched transcription factors in both CT and WT at 2W+ 11D.

Transcription factor (TF) enrichment analysis across three drought time points (2W + 5D, 8D, and 11D) revealed unique regulatory strategies in WT and CT. The results are presented in **Figures 8**–**10**.

At 2W + 5D, WT plants showed enrichment exclusively in HB-HD-ZIP. This family of homeodomain leucine zipper transcription factors has been strongly implicated in mediating drought stress tolerance by regulating leaf development, stomatal closure, and ABA-responsive gene expression (Li Y. et al., 2022). The exclusive early enrichment of HB-HD-ZIP in WT suggests a rapid and specific stress-adaptive transcriptional program consistent with ATHB-12 upregulation, suggesting a targeted ABA/stomatal regulation response. In contrast, CT plants exhibited a more diffuse response, with mild enrichment across several families including AP2/ERF, bHLH, MYB, Tify, and B3. These TFs are generally associated with various aspects of abiotic stress signaling, yet their early and simultaneous activation in CT may reflect a non-specialized and potentially inefficient response with respect to water deficit.

By 2W + 8D, WT maintained a focused enrichment of HB-HD-ZIP, reinforcing its central role. CT, however, expanded to seven TF families, with AP2/ERF-ERF and WRKY most prominent, indicating increased ethylene and pathogen/stress signaling (Kong et al., 2023) (Zhang et al., 2022). Continued enrichment of MYB and bHLH suggests ongoing growth regulation, while C2H2, C3H, and NF-YA point to further stress signaling diversity. These families regulate stress hormone signaling, detoxification, and transcriptional cascades involved in stress perception and tolerance (Wang et al., 2021) (Qian et al., 2021) (Han et al., 2020). The tight regulation of a small set of TFs in WT suggests a refined and targeted approach to stress mitigation. In contrast, CT plants showed broader TF enrichment, particularly of AP2/ERF, WRKY, MYB, bHLH, and C2H2 families. While many of these are indeed involved in drought stress responses, their concurrent overrepresentation may reflect a compensatory transcriptional response triggered by physiological damage rather than pre-emptive stress management. The activation of NF-YA and GARP in CT also points to broader developmental reprogramming, possibly at the expense of water-saving responses.

At the drought stage (2W+11D), CT plants exhibited widespread transcription factor enrichment across more than 30 TF families, including AP2/ERF, bHLH, MYB, bZIP, NAC, GRAS, TCP, and MADS-type factors. This extensive diversification likely represents a generalized stress-induced transcriptional reprogramming, suggestive of late-stage stress response rather than proactive acclimation. Conversely, WT plants maintained a moderate and selective TF profile, continuing to enrich HB-HD-ZIP, along with WRKY, NAC, bHLH, and MYB families. These TFs are well-known for their roles in abiotic stress tolerance. For example, WRKYs and NACs are implicated in ABA and jasmonic acid pathways, stomatal regulation, and senescence delay (Qiao et al., 2016) (Xie et al., 2021) (Han et al., 2023); bHLH TFs regulate ROS homeostasis and secondary metabolism; MYBs contribute to cell wall modification and osmotic adjustment (Guo et al., 2017) (Zhao et al., 2020). The persistent and coordinated activation of these TFs in WT suggests that *S. pennellii* orchestrates a more efficient and anticipatory regulatory response, minimizing the metabolic cost of stress response and preserving physiological integrity.

Taken together, these findings support the hypothesis that the drought tolerance observed in *S. pennellii* arises from an early, consistent, and focused activation of core drought-responsive TF families, particularly HB-HD-ZIP, WRKY, and NAC. These TFs likely function synergistically to enhance water-use efficiency, maintain cellular homeostasis, and regulate stress-responsive gene networks. In contrast, the cultivated *S. lycopersicum* exhibits a broader and less coordinated transcriptional response, likely reflecting susceptibility to drought-induced damage and a reliance on reactive stress signaling. These insights underscore the potential of *S. pennellii* TF networks as valuable targets for improving drought resilience in cultivated tomato through breeding or biotechnological approaches.

### 3.5 WGCNA and network analysis

**Figure 11:**
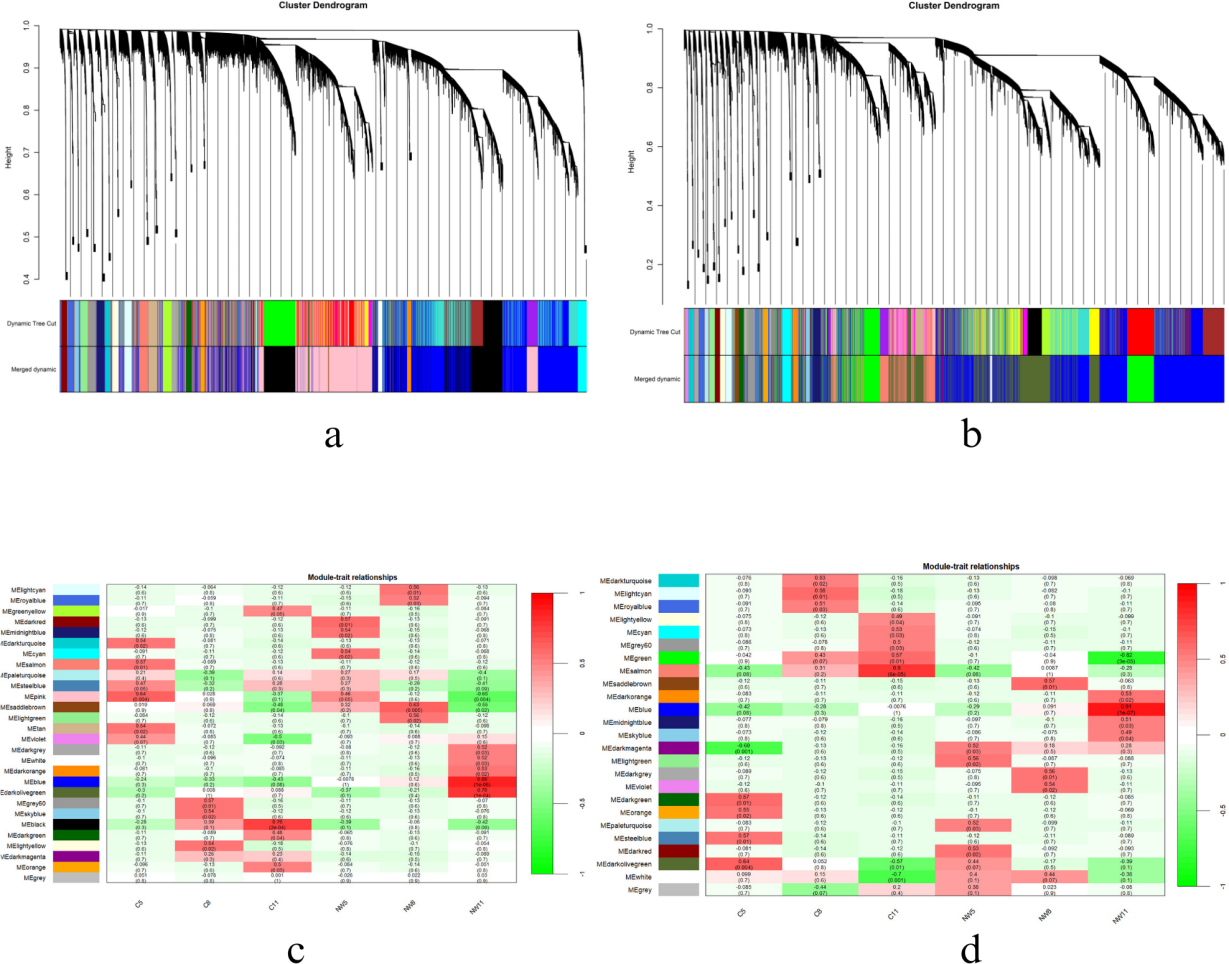
Dendrogram and significant module of 2W_11D of WT **(a, c)** and CT **(b, d)**.

To reveal the differences in gene regulation of the drought response in *Solanum* varieties with contrasting drought tolerances, weighted gene coexpression network analysis (WGCNA) was performed on 24802 genes in WT and 25650 genes in CT (**Figures 11a, c**). The vst normalized samples were clustered and outliers were removed. The soft threshold power of 9 was selected according to the preconditions of the approximate scale-free topology. Also, a module trait relationship analysis was performed using module eigengene and metadata of the two genotypes at all three days of drought stress. The analysis identified 28 distinct co-expression modules (**Figures 11b, d**) in WT and 25 modules in CT. The significant modules that positively correlated with day 5 in WT were dark red (correlation coefficient: 0.57, p-value 0.01), day 8 was saddle-brown (correlation coefficient: 0.63, p-value: 0.005), and finally with day 11 was blue (correlation coefficient: 0.88, p-value: 1e-06). Agreeably, day 5 of CT correlated with a light-green module (correlation coefficient: 0.56, p-value: 0.02), day 8 with saddle brown (correlation coefficient: 0.57, p-value: 0.01), and day 11 with a blue module (correlation coefficient: 0.91, p-value: 1e-07). Results are provided in **Supplementary Tables 3** and **4**.

Day 5 of drought significantly correlated with dark red module (1800 genes) in WT and light green module (1913 genes) in CT. Similarly, WT_8D and CT_8D correlated with saddle brown in both cases with 1617 and 1917 genes respectively. The above-mentioned numbers were obtained after conversion of Ensembl IDs to gene names using the DAVID tool. The resultant genes were later used for network creation and subsequent pathway enrichment analysis using STRING db. After applying a stringent cut-off of 0.7, the high confidence network for both WT_5D, CT_5D and WT_8D, CT_5D were constructed and analysed.

#### 3.5.1 Early drought response (2W +5D)

**Figure 12:**
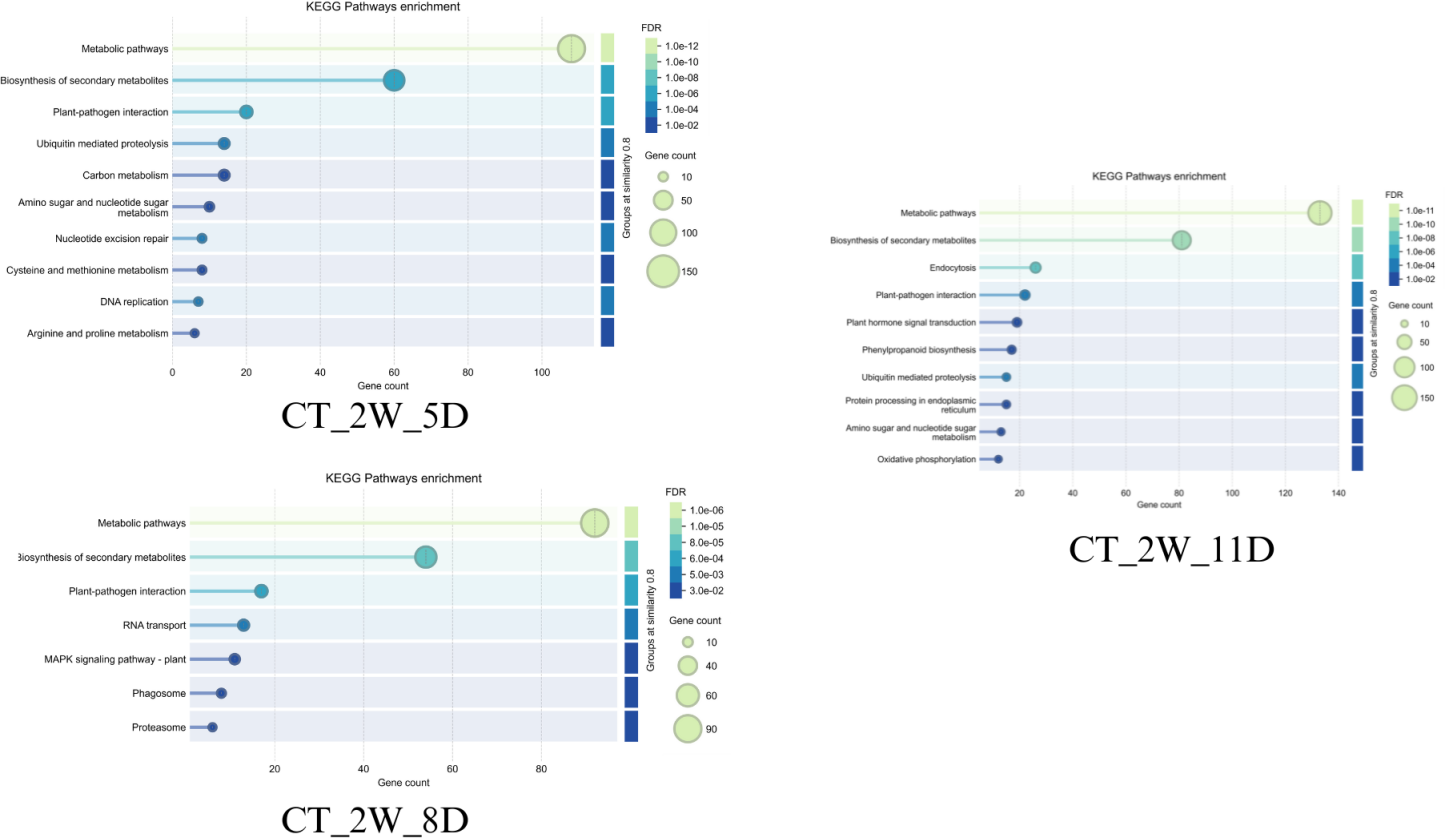
Top enriched KEGG terms of dark red (2W+5D), saddle brown (2W+8D)and blue modules(2W+11D) of WT.

**Figure 13:**
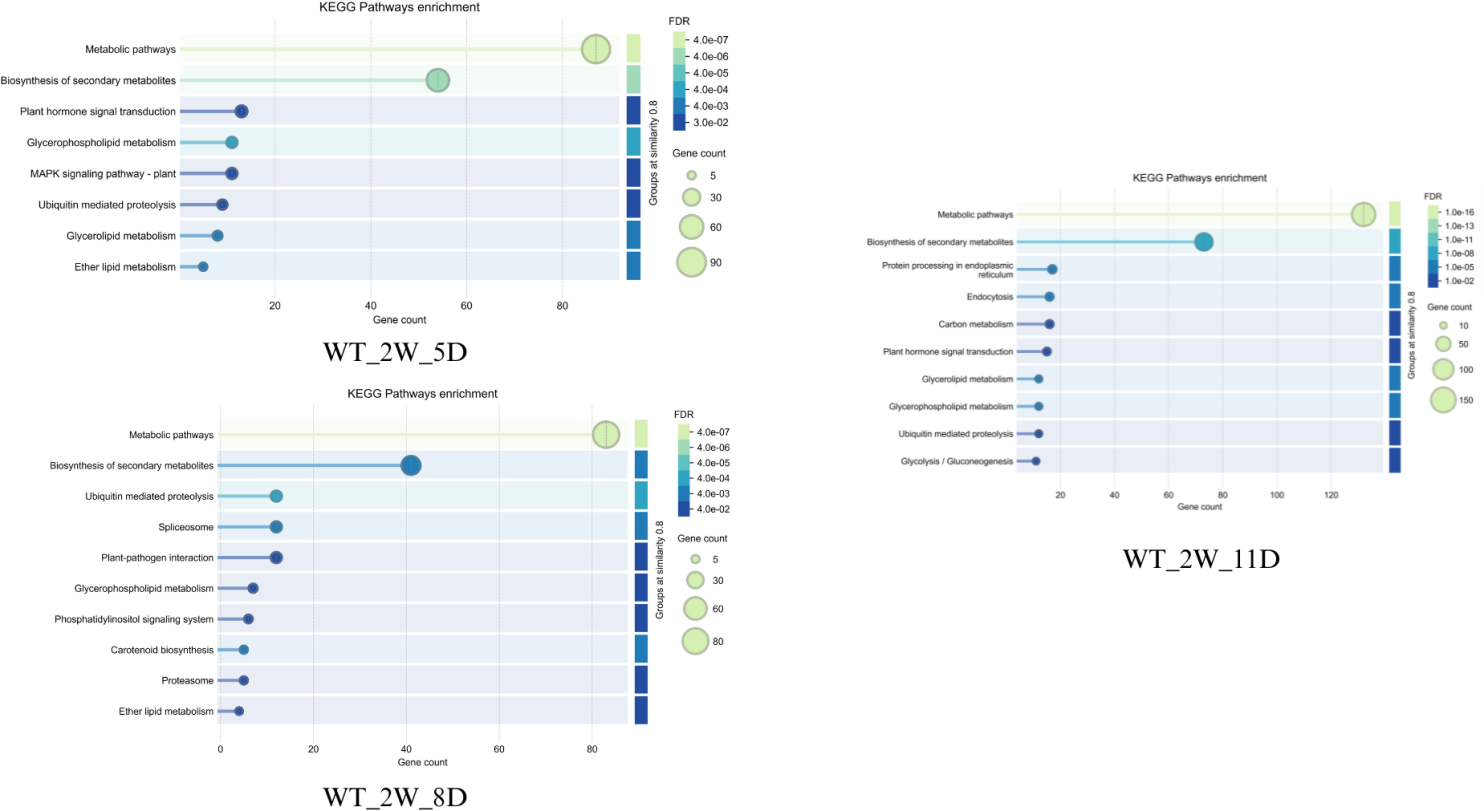
Top enriched KEGG terms of light green (2W+5D), saddle brown (2W+8D) and blue modules (2W+11D) of CT.

Both wild-type (WT_2W_5D) and cultivated (CT_2W_5D) Solanum show strong initial responses after 5D of drought (**Figures 12**, **13**). These responses include significant gene involvement (up to 90 and 150 genes, respectively) and highly significant pathways (FDR ~4e-07 for wild-type and 1e-12 for cultivated). Biosynthesis of secondary metabolites and metabolic pathways predominate in wild type, indicating an early emphasis on the synthesis of protective compounds and metabolic adaptation. Like this, cultivated Solanum gives priority to these pathways; however, it exhibits a more extensive and intense metabolic reprogramming, as evidenced by its higher gene counts (150) and lower FDR (1e-12). This discrepancy might be a result of the cultivated type’s improved genetic ability to quickly mobilise resources in response to drought stress, perhaps because of selective breeding. Furthermore, pathways such as amino sugar and nucleotide sugar metabolism, which allude to early energy and structural adjustments, are seen in cultivated Solanum which are less pronounced in wild type.

#### 3.5.2 Mid-drought response (2W +8D)

The response patterns start to differ more pronouncedly on day eight. Alongside newer pathways like spliceosome and plant-pathogen interaction (FDR ~4e-03, 30–60 genes), wild-type Solanum (WT_2W_8D) retains high significance (FDR ~4e-07) in metabolic pathways with ~80 genes, indicating a shift towards cellular stress management and possible secondary stress responses (**Figures 12** and **13**). Cultivated Solanum (CT_2W_8D) on the other hand exhibits a slight decrease in significance (FDR ~1e-08) and gene count maximum (90), with metabolic pathways and biosynthesis of secondary metabolites continuing to be central, with RNA transport and MAPK signalling pathway - plant (FDR ~1e-04 to 1e-02, 10–40 genes) serving as supplements. This may indicate a more sophisticated adaptation as the cultivated type introduces signalling and transport mechanisms while consolidating its metabolic response. The wider inclusion of stress-related pathways in the wild-type might suggest a less specialised response, potentially due to its natural variability and lack of selective optimization.

#### 3.5.3 Late drought response (2W +11D)

Both types increase their responses at 11D, but they do so with different emphasis (**Figures 12**, **13**). The systemic metabolic and protein homeostasis effort under extreme stress is indicated by the wild-type Solanum (WT_2W_11D) exhibiting metabolic pathways with an exceptionally low FDR (1e-16) and ~120 genes, as well as biosynthesis of secondary metabolites and protein processing in endoplasmic reticulum (FDR ~1e-08 to 1e-06, 50–100 genes). In cultivated Solanum (CT_2W_11D), metabolic pathways (FDR ~1e-11, ~120 genes) are also given priority, but phenylpropanoid biosynthesis and oxidative phosphorylation (FDR ~1e-04 to 1e-02, 10–50 genes) are introduced, indicating an emphasis on energy metabolism and secondary metabolite production. As a result of domestication for resilience, the cultivated type may have a genetically enhanced capacity to sustain energy production and protective compound synthesis, as evidenced by its lower FDR (1e-11 vs. 1e-16) and additional pathways. The wild-type lower FDR and focus on protein processing could indicate a natural strategy to maintain cellular integrity under extreme conditions.

Taken together, wild-type Solanum (WT) may exhibit better drought tolerance than the cultivated type (CT) due to its adaptive strategies. Early on, WT efficiently activates biosynthesis of secondary metabolites and metabolic pathways matching CT’s response with fewer genes. By day 8, WT sustains high significance (FDR ~4e-07) and diversifies into spliceosome and plant-pathogen interaction, reflecting broader stress management. On day 11, focus on protein processing in the endoplasmic reticulum to ensure cellular integrity under extreme stress. This metabolic flexibility and stability likely make WT more resilient to prolonged drought compared to more specialized responses of CT.

Focusing on day 11, a significant correlation was observed with the blue module on both species (**Figure 11**). In the blue module corresponding to WT_11, 3532 genes were grouped, and, in the CT,_11 blue module, 4607 genes were present. The ensemble IDs were converted to gene names using DAVID and the resulting 2330 genes in WT_11 and 3007 genes in CT_11 were utilized for network creation. Using a stringent confidence score of 0.7, a network with 418 nodes was created for WT_11 and 1464 for CT_11.

**Figure 14:**
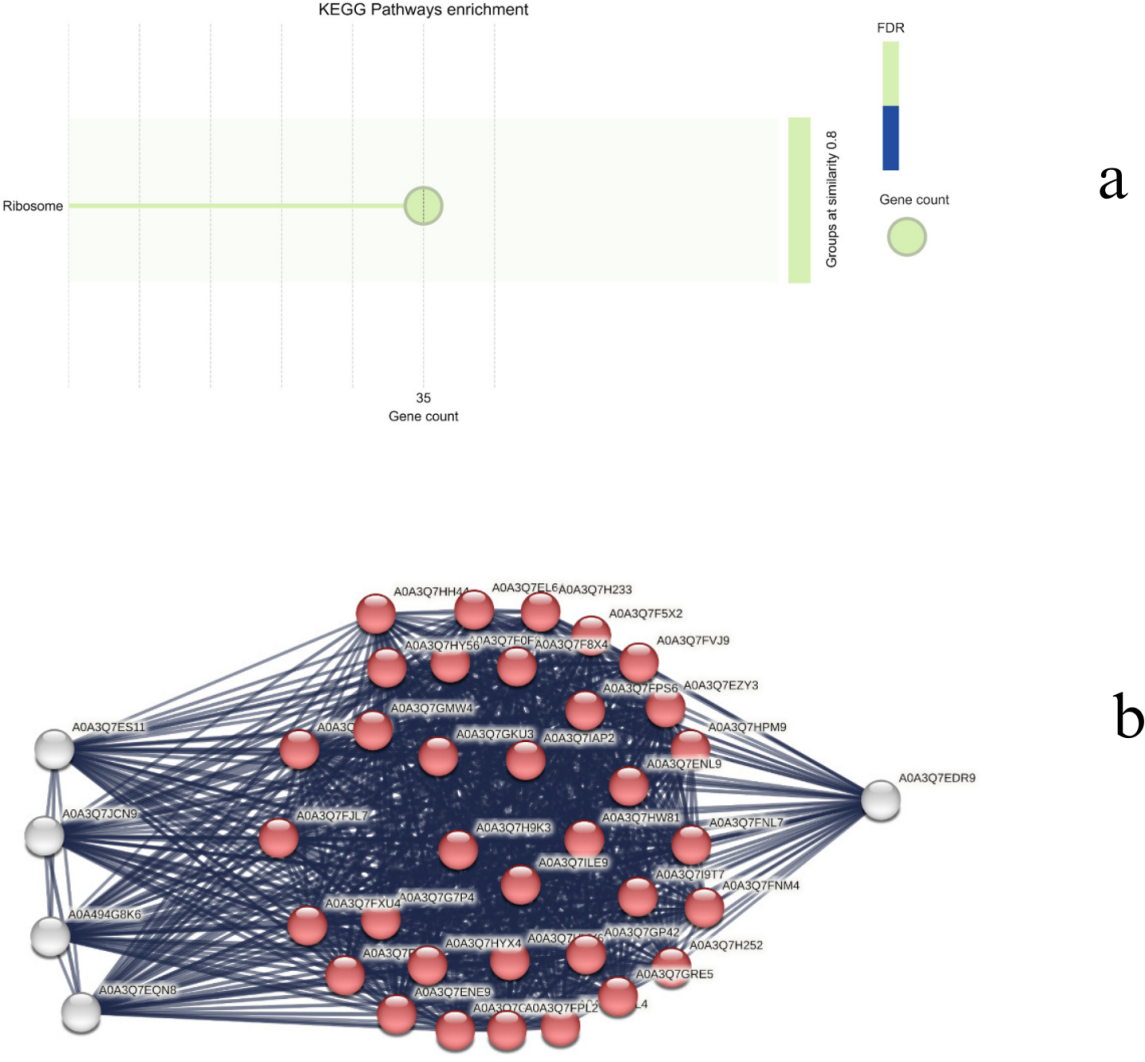
**(a)** Top enriched KEGG terms and **(b)** network representation of the top 40 hub genes in CT.

**Figure 15:**
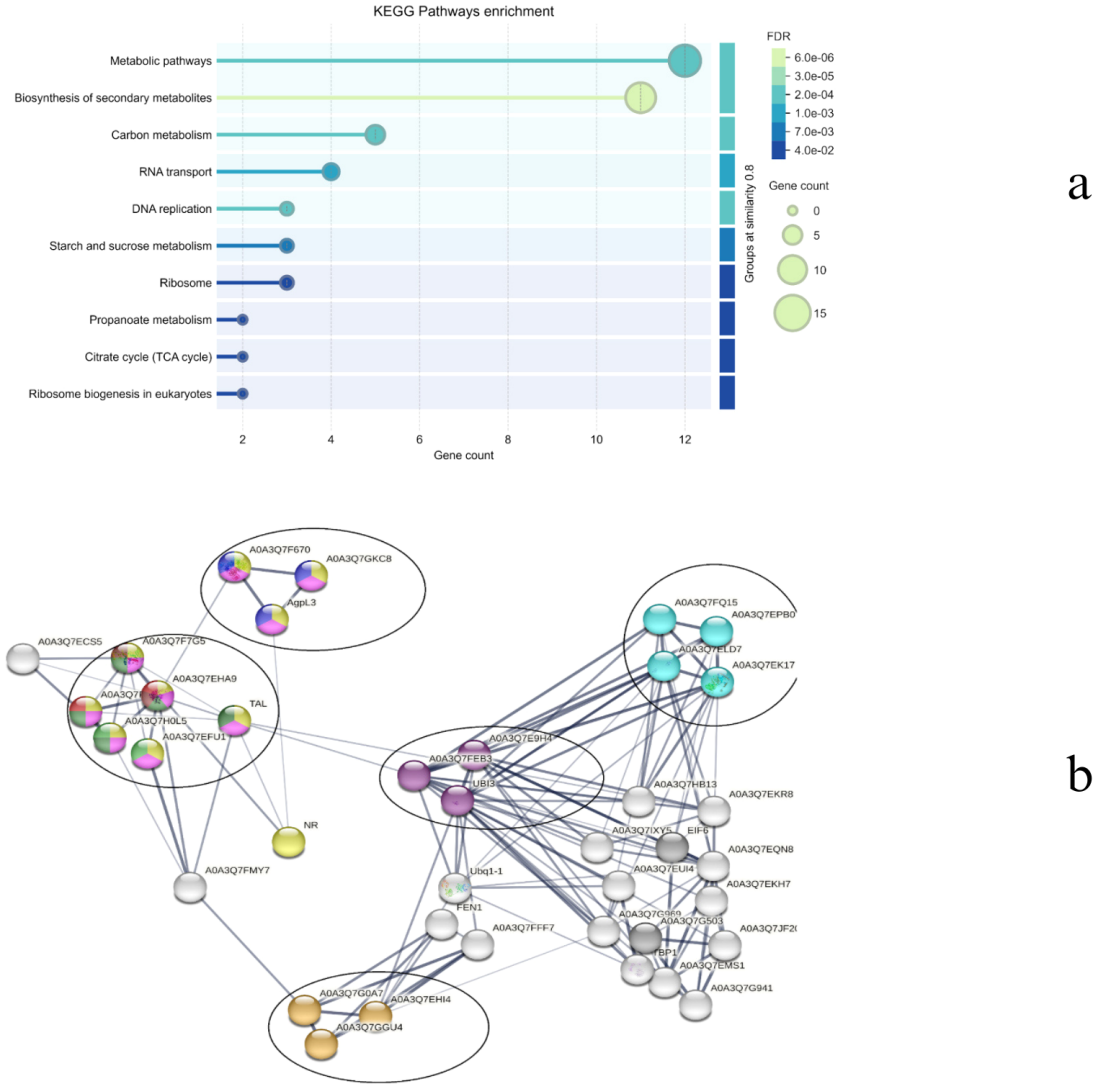
**(a)** Top enriched KEGG terms and **(b)** network representation of the top 40 hub genes in WT.

The top forty genes were identified using the degree centrality of the Cytoscape network analyzer plugin and were analyzed to determine the major genes involved in drought response in both species. While the majority of the hub genes (~35) in CT belong to the ribosomal pathway (**Figures 14a, b**), the WT hub genes are diversified into different pathways, including metabolic pathways, biosynthesis of secondary metabolites, carbon metabolism, RNA transport, DNA replication, starch and sucrose metabolism, ribosome, propanoate metabolism, citrate cycle, ribosome biogenesis, and glyoxylate and dicarboxylate metabolism (**Figures 15a, b**). Apart from the common ribosomal and cell function genes, A0A3Q7GKC8 (UTP–glucose-1-phosphate uridylyltransferase, UGPase), A0A3Q7F670 (Sucrose synthase), and AgpL3 (Glucose-1-phosphate adenylyltransferase), which are involved in starch and sucrose metabolism, as well as A0A3Q7FP95 (Malate synthase) and A0A3Q7EHA9 (Citrate synthase) of the glyoxylate and dicarboxylate metabolism, are identified as major hub genes in the WT drought response.

UGPase aids in the synthesis of UDP-glucose, a key precursor for both starch and cell wall polysaccharide biosynthesis (Meng et al., 2009). During drought stress, plants often shift from growth-related processes to stress tolerance mechanisms. The ability to produce and store carbohydrates in the form of starch is vital for maintaining energy reserves during periods of water scarcity. Additionally, UDP-glucose is involved in cell wall modifications, which can be important for maintaining cell turgor pressure and preventing water loss during drought. By regulating the balance between starch and sucrose, UGPase can help plants manage osmotic pressure under drought conditions. Role of UGPase expression is modulated during abiotic stress that is discussed in *Arabidopsis* (Ciereszko et al., 2001). Sucrose synthase plays a pivotal role in sucrose metabolism, a key pathway in plant carbohydrate allocation, particularly in the conversion between sucrose and glucose or fructose. Sucrose is a major form of sugar transported throughout the plants, and under drought stress, plants rely heavily on sucrose to provide energy for stress responses and to regulate osmotic potential. Study reported the regulatory role of sucrose synthase in pollen viability under heat and drought stress in maize (Li H. et al., 2022). Similarly, in *Cucumis sativus* (Chen et al., 2021), drought tolerance was improved by regulating sucrose metabolism. Although studies regarding the role of sucrose metabolism during drought is explored in few plants, the explicit role of sucrose synthase is relatively unexplored and therefore provide scope for further exploration. AGPL3 is involved in the biosynthesis of ADP-glucose, a precursor for starch synthesis. Starch is a crucial carbohydrate storage form that can be mobilised to provide energy during periods of stress, such as drought. Overexpression of AGPL3 has been linked to increased starch accumulation and potentially enhanced drought tolerance in rice (Prathap and Tyagi, 2020). Similarly, downregulation of AGPL3 was reported during drought stress in *Persea americana* (Zhang et al., 2023) and *Triticum aestivum* (Cui et al., 2019).

Malate synthase is an enzyme in the glyoxylate cycle, which is involved in the conversion of acetyl-CoA into carbohydrates. The glyoxylate cycle is important in seedling germination and under conditions of stress, as it allows plants to produce sugars from fats or lipids. The activity of malate synthase can help plants adapt to drought by enabling more efficient use of energy reserves and ensuring the continued production of metabolites required for stress responses (such as osmolytes like proline and sugars). Overexpression of *Ricinus communis* L. malate synthase is reported to enhance the seed tolerance to abiotic stress during germination (Brito et al., 2020). Citrate synthase is involved in the citric acid cycle (TCA cycle), which is essential for cellular respiration and energy production. The TCA cycle generates ATP, which is critical for maintaining cellular function during stress. Under drought conditions, mitochondrial respiration becomes even more crucial as the plant reallocates resources to maintain metabolic functions and osmotic balance. Few studies have reported the effect of citrate synthase on the plants, for example, thyme (Ashrafi et al., 2018), *Arabidopsis thaliana* (Koyama et al., 2000) and *Cynanchum thesioides* (Zhang et al., 2021).

Taken together, these enzymes help to regulate energy production, starch synthesis, and osmotic balance, enabling plants to conserve resources and adapt to water stress. By optimizing carbohydrate storage and metabolic processes, they provide better plant survival under drought conditions, making them potential targets for improving drought resilience in cultivated tomatoes *Solanum lycopersicum* (CT).

## 4 Conclusions

The present study analyzed RNA-seq data at three-time points from two *Solanum* varieties exhibiting contrasting drought tolerance to gain comprehensive insights into the genes and pathways involved in regulating drought stress tolerance. Distinct adaptive strategies between the two varieties were revealed through pathway, TF, and WGCNA analysis of the identified DEGs. A total of 5 genes in the WT and 11 genes in the CT were consistently expressed across all three time points. Wild varieties exhibit superior drought tolerance through early activation of osmoprotective mechanisms (e.g., proline accumulation via proline dehydrogenase downregulation), sustained transcriptional regulation by HB-HD-ZIP transcription factors. The blue module (WGCNA) was identified to be significantly correlated with day 11 of drought in both species and further GO and pathway analysis of these genes revealed differences in drought response in both species. Network analysis revealed several hub genes in WT, such as sucrose synthase, glucose-1-phosphate adenylyltransferase, malate synthase, and citrate synthase, which may contribute to enhanced metabolic flexibility and energy production under stress. In contrast, CT showed limited response diversity, with ribosome-related pathways being predominantly enriched, potentially indicating a prioritization of growth and development over stress adaptation. The identified hub genes represent novel targets for enhancing drought tolerance in *Solanum lycopersicum*. These genes can be experimentally validated through approaches, such as gene expression profiling, functional assays, and genome editing to confirm their roles in stress adaptation. Their successful integration into breeding programs or genetic engineering efforts provides a promising pathway for developing drought-resilient *Solanum* varieties, contributing to sustainable crop production under water-limited conditions.

Overall, this study highlights the similarities and differences adopted by two *Solanum* species during drought stress. Although few similarities are spotted, noticeable differences in drought response strategies can be observed in wild and cultivated tomatoes. The multifaceted and efficient response of WT plants offer valuable insights for improving drought tolerance in cultivated varieties. Understanding the specific roles of unique TF families and associated pathways can inform breeding programs to develop more drought-resistant crops, ensuring food security in water-scarce periods.

## Data Availability

The original contributions presented in the study are included in the article/**Supplementary Material**. Further inquiries can be directed to the corresponding author.
